# Perceptuomotor skill acquisition in a solo manual ball-and-beam task with varying accuracy requirements

**DOI:** 10.3389/fpsyg.2024.1436099

**Published:** 2024-08-29

**Authors:** Marijn S. J. Hafkamp, Remy Casanova, Reinoud J. Bootsma

**Affiliations:** Institut des Sciences du Mouvement, Aix-Marseille Université, CNRS, Marseille, France

**Keywords:** perceptuomotor skill, skill acquisition, motor control, motor learning, dynamical systems, behavioral dynamics, speed-accuracy trade-off

## Abstract

In the manual ball-and-beam task, participants have to control a ball that is rolling continuously on a long and hand-held beam. Since the task can be performed individually, in a solo action setting, as well as collaboratively, in a (dyadic) joint action setting, it allows us to investigate how joint performances arise from individual performances, which we investigate in a series of interrelated studies. Here we focused on individual skill acquisition on the ball-and-beam task in the solo action setting, with the goal to characterize the behavioral dynamics that arise from learning to couple (ball motion) perception and (beam motion) action. By moving a beam extremity up and down to manipulate the beam’s inclination angle, the task’s objective was to roll the ball as fast as and accurately as possible between two indicated targets on the beam. Based on research into reciprocal aiming tasks, we hypothesized that the emergent dynamics of the beam’s inclination angle would be constrained by the size of the targets, such that large targets would evoke a continuous beam movement strategy, while small targets would lead to a discrete beam movement strategy. 16 participants individually practiced the task in two separate six-block sessions. Each block consisted of one trial per target-size condition (small, medium and large). Overall, the number of target hits increased over trials, due to a larger range of motion of the beam’s inclination angle, a stronger correlation between the ball and beam motion and a smaller variability of the beam motion. Contrary to our expectations, target size did not appreciably affect the shape of the beam movement patterns. Instead, we found stable inter-individual differences in the movement strategies adopted that were uncorrelated with the number of target hits on a trial. We concluded that multiple movement strategies may lead to success on the task, while individual skill acquisition was characterized by the refinement of behavioral dynamics that emerged in an early stage of learning. We speculate that such differences in individual strategies on the task may affect the interpersonal coordination that arises in joint-action performances on the task.

## 1 Introduction

In the manual ball-and-beam task introduced by Hafkamp et al. (2023), participants have to control the motion of a ball that is rolling on a long, hand-held beam. To do so, they have to adjust the inclination of that beam continuously, so as to use the force of gravity to their advantage. The goal of this novel perceptuomotor task is to roll the ball as fast and accurately as possible between two indicated targets on the beam, with the instruction to hit (i.e., reverse direction of ball motion within) these targets as often as possible within a pre-defined timespan (e.g., 2 min). While its goal is easy to understand for participants, the task itself is quite challenging. It implies an indirect control of the ball’s motion via manipulation of the beam’s inclination. What makes it particularly interesting is that the task can be performed alone (i.e., in a solo action setting) and in collaboration with someone else (i.e., in a dyadic joint action setting). In the solo action setting, one end of the beam is statically supported while the other extremity is manipulated by the agent. In the joint action setting, the two extremities are manipulated by different agents, meaning that a stable mode of interpersonal coordination is required to control the rolling ball on the beam.

A first study on this task (Hafkamp et al., 2023) demonstrated that the emergence of interpersonal coordination was dependent on the (solo) task experience of the two individuals involved. When both players were unexperienced with respect to the task, they typically moved the beam in a sequential, alternating fashion, each player freezing their motion while the other moved. In contrast, when both players had received individual practice prior to the joint action session, they moved the beam concurrently and in opposite directions. This effect suggests that the mode of interpersonal coordination that arises in a joint action setting, is somehow dependent on the performances of individuals in solo action setting. Apparently, experienced ball-and-beam players bring something to the interaction that unexperienced players do not. There is, in other words, a transfer of skill that affects the outcome of the joint action performance. In a series of studies, we set out to investigate the nature of this transfer, with the goal to understand how a coordinated team can arise from a collection of individuals (Baron, 2007). As a first step, the current contribution aims to identify the pertinent characteristics of individual performances in the solo ball-and-beam setting. How does a player learn to control the ball on this task? And is this learning process the same for all individuals, or do qualitative differences in their performances emerge over practice? In a follow-up contribution, we will explore which aspects of these individual performances are transferred to a joint action setting and which aspects are left behind in the interaction.

In our previous study (Hafkamp et al., 2023) we observed that individual practice in the solo ball-and-beam setting resulted in a significant increase in the number of target hits per trial. In part, this increase was due to a higher average speed of the ball, caused by an increasing range of motion of the beam’s inclination angle. This, however, was not the only factor that contributed to the improvement in performance over trials. We also found that, notwithstanding the increase in ball speed, the consistency of the ball motion around the targets improved over practice. Together, these findings indicated that task practice improved a participant’s ability to control the ball motion. In other words, it suggested that participants had learned how to coordinate the motion of the beam with the motion of the ball on that beam. To achieve such ‘ball-beam coordination’, a player needs to learn how to couple its action of lifting and lowering the beam with its perception of the ball rolling between the two targets. As such, the challenge for the player lies in relating the beam’s motion to the pertinent information that specifies the motion of the ball (see for instance Jacobs et al., 2012, for perception-action coupling in pole balancing). This continuous and reciprocal perception-action coupling (Bootsma and van Wieringen, 1990; Bootsma, 1998) ultimately gives rise to an observable dynamics of the ball-beam system, which, following Warren (2006), we refer to as its behavioral dynamics. The primary goal of the current study was to characterize this behavioral dynamics. Which patterns of ball and beam motion arise from the perception-action coupling in this ball-and-beam task? And does the same behavioral dynamics emerge for all individuals or does practice lead to noticeable differences between players?

To understand which behavioral dynamics might arise, it is helpful to take a closer look at dynamical properties of the ball-and-beam task as a physical system (Bolívar-Vincenty and Beauchamp-Báez, 2014; Debono and Bugeja, 2015). For starters, the ball is set into motion by the driving (tangential) component of the force of gravity, with the instantaneous translational acceleration of the ball thus being determined by the inclination angle of the beam. Deceleration of the ball is caused by that same (tangential component of the) force of gravity –now working in opposite directing– but also depends on the contributions of non-linear resistive forces that decelerate the ball at the same time. Thus, by moving the beam extremity up and down, agents accelerate and decelerate the ball via a complex, non-linear and gravity-mediated relationship to the ball. The behavioral question is how a player learns to deal with this particular form of indirect control, in which the relation between one’s own movements and those of the to-be-controlled object is yet to be discovered.

Theoretically, the physical properties of the system allow for different strategies of moving the beam, almost all of which would result in continuous rolling of the ball between the targets. One possibility, for instance, is to move the beam in a continuous and harmonic manner, which would be reflected in a constant phase progression of the beam inclination throughout a cycle. Another option would be to accelerate and decelerate the ball in a more discrete fashion, by slowing down or even temporarily stopping the motion of the beam at specific phases in its cycle or by adding corrective sub-movements to each cycle of motion. Due to the ball’s translational and rotational inertia, such local stops or deviations from harmonicity in beam motion would not (immediately) prevent the ball from continuing to roll. In other words, the system’s physical properties seem to provide room for strategic variability on the part of the agent, that is, multiple task solutions are hypothetically possible. For this reason, we expected that the behavioral dynamics that would arise in the performance of the task would be as much revealing about the underlying perception-action coupling, as about of the physical characteristics of the system.

In spite of the novelty of the continuous ball-and-beam paradigm (but see Huang et al., 2006 for a study on a discrete version), the more general task of reciprocally moving an object between two targets has been studied extensively in the domain of perceptuomotor control (e.g., Mottet and Bootsma, 1999; Billon et al., 2000; Mottet et al., 2001; Bootsma et al., 2004; Bongers et al., 2009; Huys et al., 2010; Valk et al., 2019). Usually, the object in question is an end-effector, such as the tip of the finger or a hand-held stylus, and the instruction is to move that end-effector as fast and accurately as possible between two targets. Research into such tasks has revealed that the behavioral dynamics of these end-effectors are strongly constrained by the precision requirements of the task (Mottet and Bootsma, 1999; Mottet et al., 2001; Bootsma et al., 2004; Bongers et al., 2009). When the targets are large, implying low demands on the accuracy of the movement, agents move their end-effector in a smooth and continuous manner between the targets. However, when the target size is decreased, agents adopt a more discrete strategy by slowing down their motion in the vicinity of the target zones, ultimately even coming to full stops. Although there is some debate about whether this change in dynamics reflects a transition between two fundamentally different strategies (Huys et al., 2010), or a more gradual adaptation of parameters within the same dynamical structure (Mottet and Bootsma, 1999; Bongers et al., 2009), it is clear that the change is accompanied by a lengthening of the deceleration time. This leads to a longer movement time from target to target, which accounts for the well-known and highly robust speed-accuracy trade-off in the control of our movements (Fitts, 1954; Fitts and Peterson, 1964).

In applying these principles to our ball-and-beam task, we distinguished between the dynamics of the ball and the dynamics of the beam. Due to the inertial properties of the ball, we expected the ball to roll continuously back-and-forth at all times, regardless of the size of the targets or the amount of expertise of the participants. In contrast, we hypothesized that the dynamics of the beam would change as a function of the task’s accuracy constraints. More specifically, we expected that the strategy of the lifting and lowering the beam extremity would become more discrete (as opposed to continuous) when the size of the targets was decreased. Thus, we hypothesized that the behavioral dynamics in this task –reflected in the motion of the beam in relation to the position of the ball– would depend on the task’s accuracy demands, similar to what has been reported in reciprocal aiming paradigms. To test this hypothesis, we studied individual participants practicing the (solo) ball-and-beam task under three different target size conditions: small targets, medium targets and large targets. With respect to the effect of practice, we speculated that the beam’s behavioral dynamics would become more consistent and more continuous over trials, especially in the largest target-size condition, reflecting the process of learning to control the ball on the beam.

## 2 Materials and methods

### 2.1 Participants

A group of 16 students and junior staff members from Aix Marseille University (7 women, 9 men, with an average age of *M* ± *SD*: 23.3 ± 2.9 years) participated voluntarily in this study. None of them had any experience on the task. All participants were free from known motor impairments and reported normal or corrected-to-normal vision. Before the start of the first experimental session the participants were informed about the aim and procedure of the experiment. All participants provided written informed consent before participating in the study. The study was approved by the French National Ethics Committee for Research in Sports and Movement Sciences (CERSTAPS) and conducted according to University regulations and the Declaration of Helsinki.

### 2.2 Task and procedure

The task equipment consisted of a standard golf ball (diameter 4.3 cm, weight 46 g) that could roll over a 2-m long, 3 by 3 cm V-shaped iron beam, with tubular (length 54 cm, diameter 3 cm) handles perpendicularly attached to its extremities. Participants’ task was to roll the ball as fast and accurately as possible back-and-forth between two targets clearly visible on the beam. Participants received one point for every time that the ball reversed its direction of motion within a target’s boundaries, with the objective of achieving as many points as possible within every 2-min trial. With both target centers located 50 cm from the beam’s midpoint, the center-to-center inter-target distance was 100 cm in all trials. The size of both targets varied from trial to trial and was either 6, 12 or 24 cm. In the following, we will refer to these targets as small, medium and large targets. Bimanually holding one handle allowed a participant to lift or lower the beam’s extremity and thereby change its inclination angle so as to accelerate or decelerate the ball. The second of the two handles was attached to a hinge on a static pole, the height of which was adjusted to the participant’s hip height.

Participants performed two sessions of individual practice in the solo action setting of the ball-and-beam task that were to be completed on two different days, with one or two days between the sessions. A session began with a short warm-up trial of approximately 1 min, during which participants were given the chance to familiarize themselves with the task. The familiarization was followed by six blocks of practice per session. Each of these six blocks consisted of three trials: one trial with small targets, one with medium targets and one with large targets. The three conditions within a block were presented in a pseudo-randomized order over blocks, such that all possible orders of conditions were presented once during a session. After every two blocks of trials, participants were given a short break of 2–3 min. Given the two sessions of practice, each participant performed a total of 12 blocks of practice, corresponding to 36 individual trials. We note that in a follow-up study, these 16 participants were paired into 8 dyads who then practiced of the ball-and-beam task in a joint action setting. Results of this latter study will be reported separately.

At the start of each 2-min trial, the ball was placed in the middle of the beam. Verbal signals from the experimenter signaled the beginning (‘Go’) and the end (‘Stop’) of a trial. After each trial, participants received verbal feedback on their score, based on experimenter observation. Participants were regularly encouraged to try to improve their score on each new trial, in order to reach the highest score possible in each target size condition. Participants competed indirectly with the other players via a leaderboard that expressed the high scores of the other participants on each target size condition. Moreover, small (delicacy) prizes were awarded to those with the highest scores. All of this was in function of motivating the participants to perform optimally on each new trial.

### 2.3 Data acquisition and analysis

All sessions took place in a darkened room. To capture the motion of the beam, small reflective markers were attached to each handle through a thin, rigid and 10-cm long aluminum rod. In order to capture the motion of the ball, we chose to use a bright yellow color for the ball. Participants wore dark clothes and black gloves to ensure sufficient contrast with the ball and the two beam markers on the beam. All trials were filmed with a GoPro9 camera that was positioned at a 2-m perpendicular distance from the midpoint of the beam. The camera, set in linear lens mode so as to obtain minimal image deformation, was placed on a tripod at a 1-m height. All trials were recorded with a (recalculated) sample frequency of 29.97 Hz and a resolution of 2.7 K.

Video recordings of all trials were imported into Kinovea,^1^ which was used to track the two-dimensional (X, Y) motion of the ball and the two reflective markers on the beam. X and Y position time series were then analyzed using MATLAB. All data were first filtered using a fourth-order low pass Butterworth filter (cut off frequency of 3 Hz) and trimmed to the 120-s trial duration. The ball’s trajectory was subsequently transformed from motion in a two-dimensional space to one-dimensional motion along the beam’s longitudinal axis. To do this, we calculated the ball’s motion along the virtual vector between the two beam markers, with the inter-marker distance used for calibration. From the trajectories of the two markers on the beam we derived a single inclination angle at each time step by taking the inverse tangent (atan) of the X and Y coordinates of both markers. The one-dimensional time series of the ball motion, on the one hand, and the beam’s inclination angle, on the other hand, formed the basis of all further analyses in this contribution. In discussing these analyses as well as the results that follow from them, we progressively move from (1) the motion of the ball, via (2) the coordination of the ball with the beam, to (3) the motion of the beam. All analyses were conducted on all individual trials, including all different target size conditions.

To characterize the movements of the ball on the beam, we used a peak finding algorithm to split the ball position time series into separate half cycles. We used these half cycles to count the number of target hits per trial, which we defined as a ball motion reversal within the (recalculated) target zones of each task condition. By dividing the number of target hits by the total number of half cycles on a trial, we acquired a measure of relative accuracy in the performance. We refer to this measure as the accuracy ratio on a trial. To come to a measure of absolute consistency of the ball’s motion around the targets, we also computed the effective target size on each trial. This variable was calculated by multiplying the standard deviation of the ball position peaks around the two targets by 1.96 (Welford, 1968; see also Huys et al., 2010). Lower effective target size therefore indicated a higher average ball consistency on a trial. Lastly, we derived an average of the ball speed on each trial by multiplying the ball’s average half cycle frequency by the average peak-to-peak amplitude of the ball’s half cycles.

To assess the ball-beam coordination, we first performed a cross-correlation analysis between the time series of ball position and the beam’s inclination angle in each individual trial. From these cross-correlations, we derived the maximum Pearson correlation coefficient and its time-lag (in s). We divided this time-lag by the average half cycle time to come to a relative time-lag (in %) on every trial. Subsequently, we conducted a qualitative analysis on the dynamical patterns of the ball and beam motions in each trial. In this analysis, we interpreted the phase portraits of the ball (i.e., ball velocity as a function of ball position) and of the ball-beam coordination (i.e., beam angular velocity as a function of ball position). Based on inspection of these portraits, we decided to quantify three distinguishable aspects of the underlying beam movements: the range of motion, the variability and the shape of the beam motion pattern.

The range of motion (ROM) of the beam on a trial was calculated by determining the average angular distance between the beam at maximum and minimum inclination. As such, it constituted the average peak-to-peak amplitude of the cyclical beam movement. The variability of the beam’s inclination angle on each trial was assessed with the coefficient of variation (CV). To this end, we first divided the inclination angle time series into separate cycles on the basis of the ball position peaks. We chose to use the ball’s cycles because these were more consistent in comparison to the beam’s cycles. We then time-normalized all cycles to 100 timepoints and calculated the standard deviation over all cycles at each of the timepoints. We averaged all 100 standard deviations to come to one standard deviation per trial, which we divided by the average peak-to-peak amplitude on that trial to come to the CV. Thirdly, we quantified the average shape of the beam movement by taking the ratio (shape ratio, SR) between the average absolute inclination angle and half of the average peak-to-peak amplitude of the beam motion. The SR was inspired by the shape of the histograms of the beam inclination angle on each trial.

As a final analysis, we assessed the relationship between the three beam motion variables on the one hand and three ball-level indicators of performance (target hits, ball speed and accuracy ratio) on the other hand. To do so, we computed the Pearson correlation coefficients over participants between each of these variables for all three target size conditions separately.

### 2.4 Statistical analysis

A 3 × 12 repeated measures ANOVA, with the factors Condition (3 target size conditions) and Practice (12 practice blocks), was used to analyze the following dependent variables: target hits, ball speed, accuracy ratio, ball effective target size, (relative) ball-beam time-lag, maximum ball-beam correlation coefficient, beam ROM, beam CV and beam SR. Pearson correlation coefficients were standardized using a Fisher-Z transformation before performing the analysis of variance. For all analyses the significance level was set to *α* = 0.05. Greenhouse–Geisser corrections were applied when violations of the sphericity assumption were detected. For reasons of readability, all ANOVA results were summarized in a single table. Post-hoc tests were performed using Tukey HSD. Where appropriate means and standard deviations are reported as M ± SD.

## 3 Results

### 3.1 Ball motion

#### 3.1.1 Target hits and ball cycles

**Figure 1:**
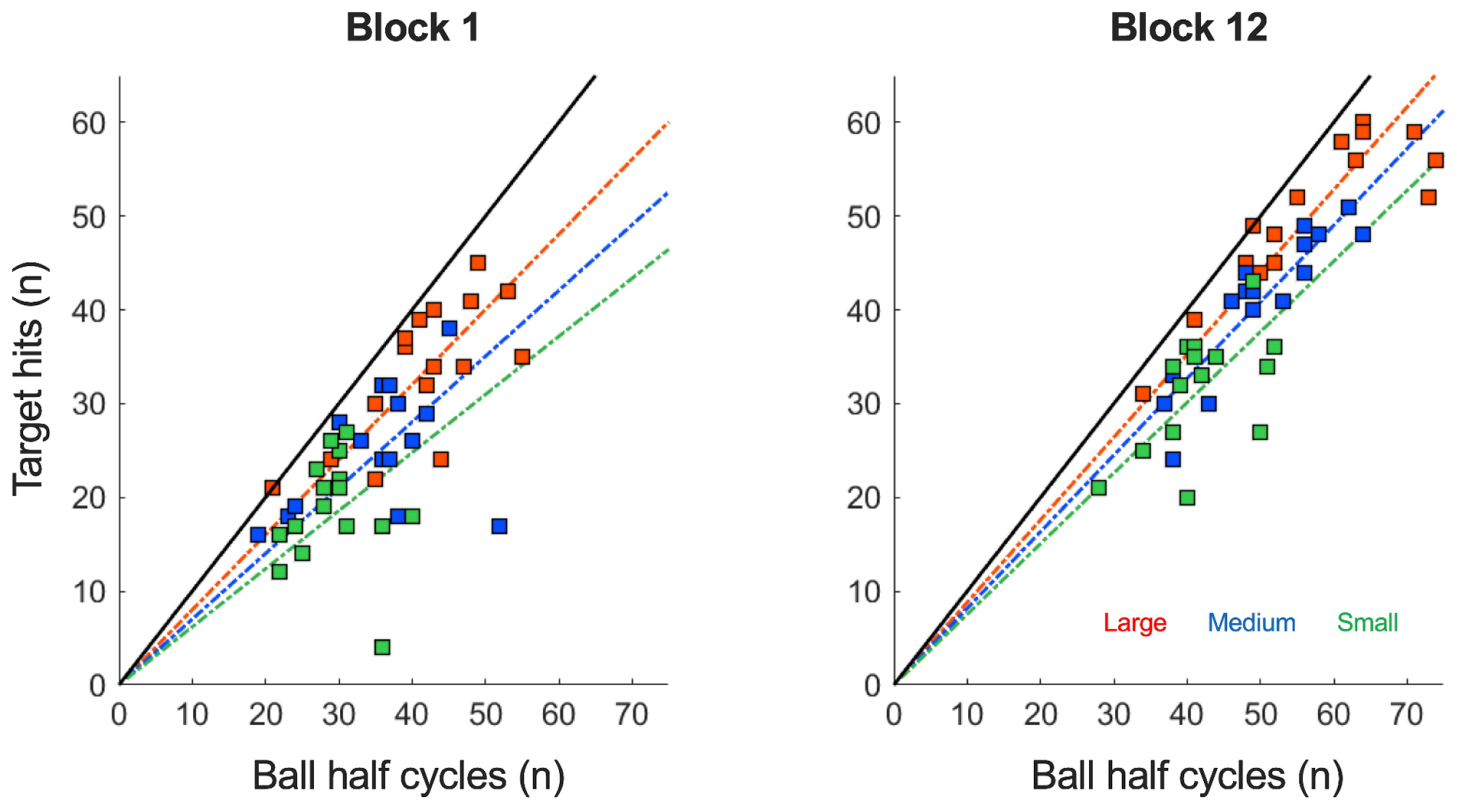
Target hits (n) as a function of ball half cycles (n) under each of the three target size conditions (small: green; medium: blue and large: red) in the trials of block 1 and 12. From block 1 to 12, the number of target hits and ball half cycles both increased significantly in all task conditions, indicating an improvement in performance. The increasing slopes in the linear regression lines from block 1 to 12 also indicate an increased accuracy of the performance in all three target size conditions.

**Figure 2:**
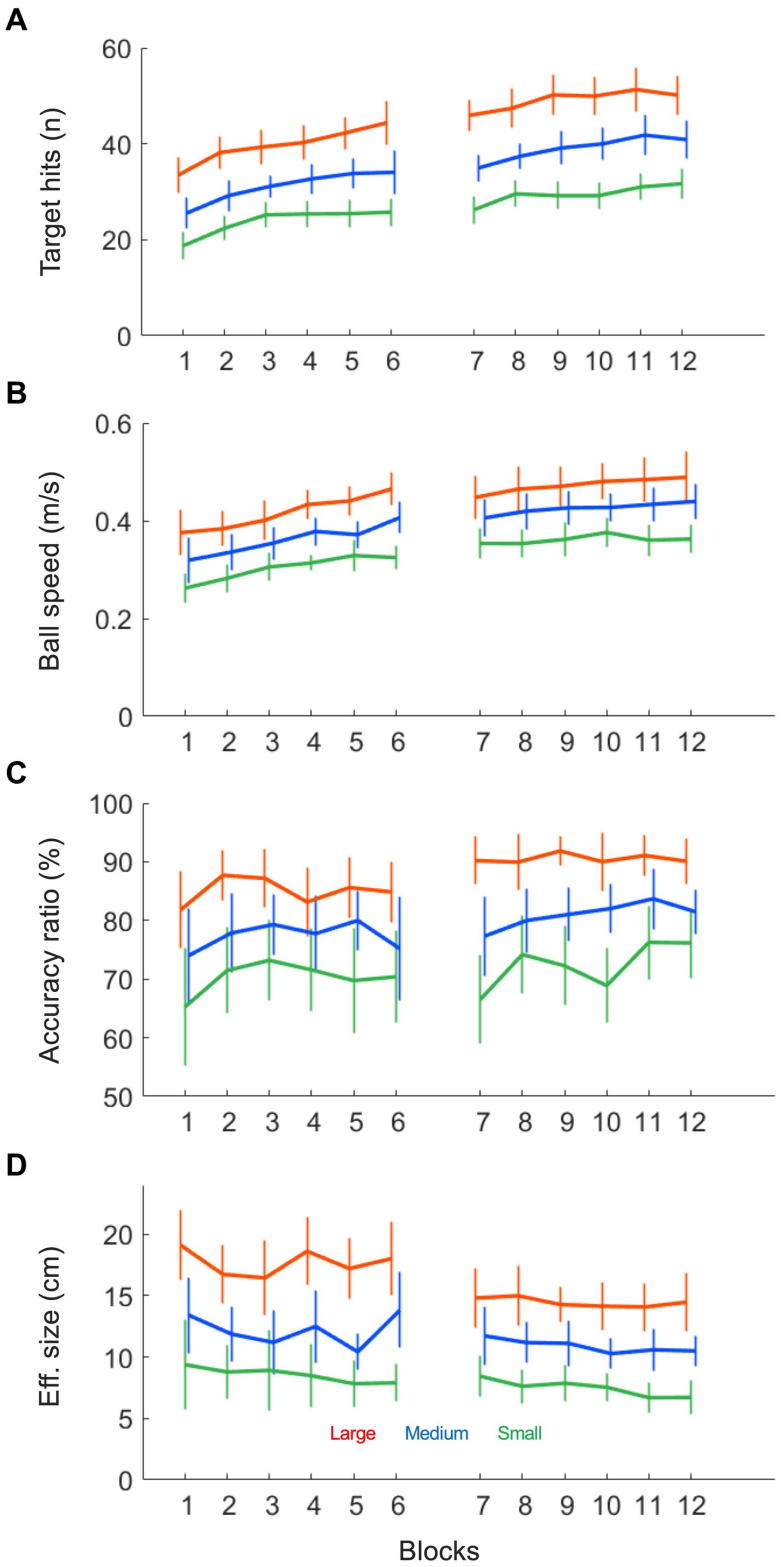
Ball motion variables as a function of practice blocks under each of the three target size conditions (small: green; medium: blue and large: red). **(A)** The number of target hits (n) increased over practice in all conditions and was higher in larger target size conditions. **(B)** The average ball speed (m/s) increased over practice in all conditions and was higher in larger target size conditions. **(C)** The average accuracy ratio (%), indicating the ball motion accuracy, increased over trials and was higher in larger target sizes. **(D)** The average effective target size (cm), indicating ball motion consistency, decreased over trials and was lower in smaller target sizes.

As can be seen in Figure 1, participants enhanced their performance from block 1 to 12, as indicated by a significant increase in the number of target hits over practice (*F* (4.43, 66.42) = 68.72, *p* < 0.001, *η^2^_p_* = 0.82). This increase in hits was different per target size condition, such that larger targets gave rise to a stronger increases in the number of hits (*F* (22, 330) = 2.40, *p* < 0.001, *η^2^_p_* = 0.14; also see Figure 2A). More generally, larger targets allowed for more hits on a trial (*F* (2, 30) = 309.00, *p* < 0.001, *η^2^_p_* = 0.95). Furthermore, Figure 1 shows that the increase in target hits was accompanied by an increasing number of total ball half cycles. This reflected a significant increase in the average ball speed (*F* (3.20, 47.99) = 19.81, *p* < 0.001, *η^2^_p_* = 0.57) over trials, which took place in all three target size conditions (also see Figure 2B). As expected, this ball speed varied in accordance with the target size, such that a larger target induced a higher average ball speed (*F* (1.13, 16.98) = 60.28, *p* < 0.001, *η^2^_p_* = 0.80). Full results of all Condition (3) x Practice blocks (12) ANOVAS are provided in Table 1.

**Table 1:** Results of Condition (3 target sizes) x Practice (12 blocks) repeated measures ANOVAs on ten dependent variables: Four related to the ball motion (Target hits, Ball speed, Accuracy ratio and Effective target size), three related to the ball-beam cross correlation analysis (Time-lag, Relative time-lag and Maximum correlation coefficient) and three related to the beam motion (Beam range of motion ROM, Beam variability CV and Beam shape ratio SR).

	Effect	*F*	(df)	*p*	η_p_^2^
Target hits	Condition	309.00	(2, 30)	< 0.001	0.95
	Practice	68.72	(4.43, 66.42)	< 0.001	0.82
	Condition * Practice	2.40	(22, 330)	< 0.001	0.14
Ball speed	Condition	60.28	(1.13, 16.98)	< 0.001	0.80
	Practice	19.81	(3.20, 47.99)	< 0.001	0.57
	Condition * Practice	1.24	(22, 330)	0.215	0.08
Accuracy Ratio	Condition	44.11	(1.41, 21.17)	< 0.001	0.75
	Practice	2.75	(4.14, 62.16)	0.034	0.16
	Condition * Practice	1.00	(22, 330)	0.459	0.06
Effective size	Condition	98.87	(1.40, 20.96)	< 0.001	0.87
	Practice	2.75	(3.23, 48.47)	0.049	0.16
	Condition * Practice	1.61	(22, 330)	0.042	0.10
Time lag	Condition	65.79	(1.25, 0.44)	< 0.001	0.81
	Practice	10.29	(2.58, 28.67)	< 0.001	0.41
	Condition * Practice	1.61	(22, 330)	0.043	0.10
Relative time-lag	Condition	59.53	(1.18, 17.69)	< 0.001	0.80
	Practice	6.75	(2.83, 42.38)	< 0.001	0.31
	Condition * Practice	0.87	(22, 330)	0.639	0.06
Max. corr. Coef.	Condition	68.65	(1.39, 20.82)	< 0.001	0.82
	Practice	38.48	(4.77, 71.49)	< 0.001	0.72
	Condition * Practice	0.47	(22, 330)	0.981	0.03
Beam ROM	Condition	28.79	(1.08, 16.24)	< 0.001	0.66
	Practice	14.94	(2.79, 41.82)	< 0.001	0.50
	Condition * Practice	2.49	(22, 330)	< 0.001	0.14
Beam CV	Condition	43.15	(1.38, 20.62)	< 0.001	0.74
	Practice	22.26	(4.91,73.71)	< 0.001	0.60
	Condition * Practice	0.97	(22, 330)	0.509	0.06
Beam SR	Condition	52.09	(1.08, 16.25)	< 0.001	0.78
	Practice	2.20	(4.44, 66.64)	0.072	0.13
	Condition * Practice	0.53	(22, 330)	0.961	0.02

#### 3.1.2 Ball accuracy and consistency

Figure 1 also provides an insight into the accuracy of the performance, since the slope of the linear regression line through the cloud of data points is directly related to the accuracy ratio on the task (45° slope indicating 100% accuracy). In general, the slope increased slightly from block 1 to 12 (*F* (4.14, 62.16) = 2.75, *p* < 0.034, *η^2^_p_* = 0.16), indicating that over practice participants achieved more hits per total number of ball cycles. This increase in accuracy ratio (also see Figure 2C) was not significantly different between conditions (*F* (22, 330) = 1.00, *p* = 0.459, *η^2^_p_* = 0.06), but the accuracy ratio was higher when the targets were larger (*F* (1.41, 21.17) = 44.11, *p* < 0.001, *η^2^_p_* = 0.75). These findings on the accuracy of the performance were supported by analysis of the consistency of the ball’s reversal locations around the targets: Effective target size (see Figure 2D) also decreased slightly but significantly over practice (*F* (3.23, 48.47) = 2.75, *p* = 0.049, *η^2^_p_* = 0.16) and was also higher for larger target sizes (*F* (1.40, 20.96) = 98.87, *p* < 0.001, *η^2^_p_* = 0.87). In contrast to the accuracy ratio, we observed a small but significant difference in the rate of change per condition, where the effective target size decreased more for larger targets (*F* (22, 330) = 1.61, *p* = 0.042, *η^2^_p_* = 0.10).

### 3.2 Ball-beam coordination

#### 3.2.1 Cross-correlation analysis

**Figure 3:**
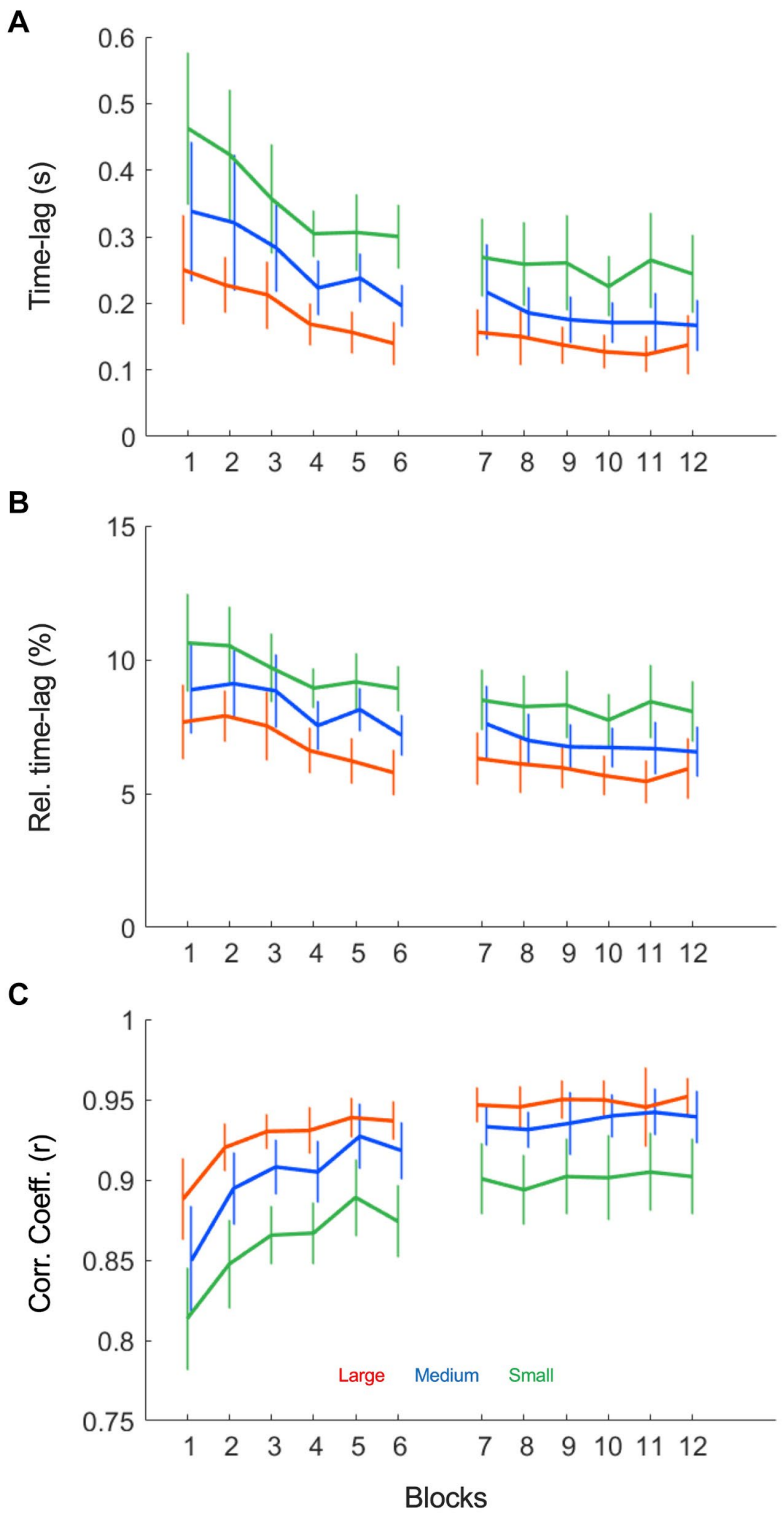
Ball position – beam inclination angle cross-correlation results as a function of practice blocks under each of the three target size conditions (small: green; medium: blue and large: red). **(A)** The time-lag (s) between ball position and beam inclination decreased over practice and was shorter for larger targets. **(B)** The relative time-lag (%) between ball position and beam inclination decreased over practice and was shorter for larger targets. **(C)** The average maximum correlation coefficient increased over blocks, most prominently in the first blocks. The correlation coefficient was higher in larger target size conditions as compared to the smaller target sizes.

To quantify the ball-beam coordination, we conducted a cross-correlation analysis of the relation between ball position and beam inclination angle. As shown in Figure 3A, maximum correlation was found at small positive time-lags, which indicated that the cyclic motion of the beam lagged behind that of the rolling ball for the majority of the cycle. This was the case in all trials. The Condition (3) x Practice (12) repeated measures ANOVA (Table 1) demonstrated that the absolute size of the time-lag shortened over practice (*F* (2.58, 28.67) = 10.29, *p* < 0.001, *η^2^_p_* = 0.41), was shorter in large target size conditions (*F* (1.25, 0.44) = 65.79, *p* < 0.001, *η^2^_p_* = 0.81) and decreased more strongly with larger target sizes (*F* (22, 330) = 1.61, *p* = 0.043, *η^2^_p_* = 0.10). Most of these differences became smaller, but nevertheless persisted, when we corrected the time-lag for the half cycle duration resulting in a relative time-lag (Figure 3B). The relative time-lag also decreased significantly over practice (*F* (2.83, 42.38) = 6.75, *p* < 0.001, *η^2^_p_* = 0.31) and was also shorter for larger target sizes (*F* (1.18;17.69) = 59.531, *p* < 0.001, *η^2^_p_* = 0.80), but the decreasing trend over practice was not significantly different between conditions (*F* (22, 330) = 0.87, *p* = 0.639, *η^2^_p_* = 0.06). The maximum correlation coefficient between ball position and beam inclination angle increased significantly over practice, most prominently in the first blocks (Figure 3C *F* (4.77, 71.49) = 38.48, *p* < 0.001, *η^2^_p_* = 0.72). Moreover, the correlation between the time series was significantly higher when targets were larger (*F* (1.39, 20.82) = 68.65, *p* < 0.001, *η^2^_p_* = 0.82), but the rate of change over practice was not significantly different between target size conditions (*F* (22, 330) = 0.47, *p* = 0.981, *η^2^_p_* = 0.03). Interestingly, the correlation coefficients leveled off after a few practice blocks, before increasing again in the first block of the second session (block 7, Figure 3C). In all subsequent blocks, however, the correlation coefficient remained stable, suggesting that a full covariation of ball position and beam inclination was not possible, desirable or both. To investigate the reason for this stagnation in more detail, we continued with a qualitative analysis of the dynamical patterns of the ball and beam system.

#### 3.2.2 Qualitative analysis

**Figure 4:**
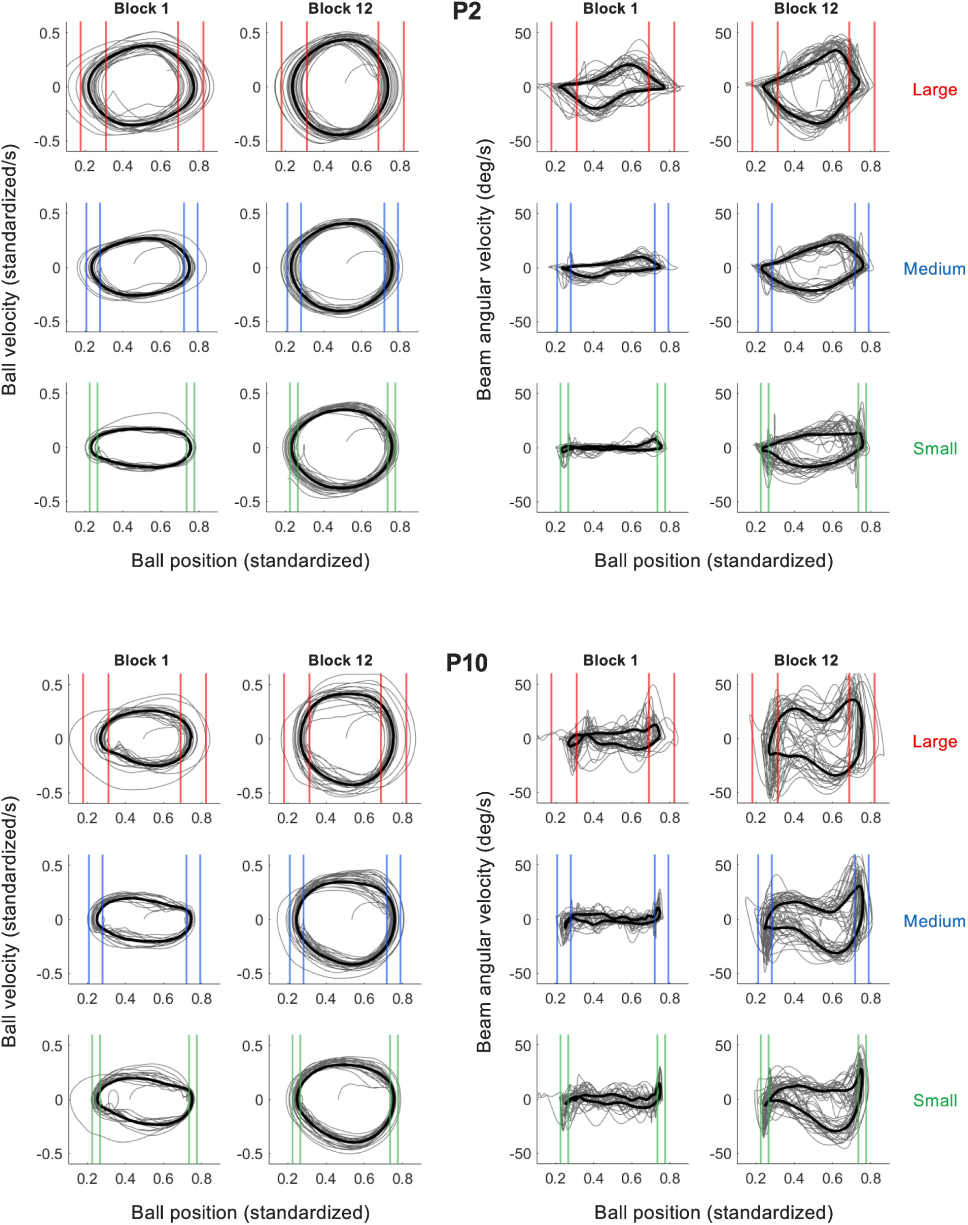
Plots of the ball velocity as a function of the ball position (left) and the beam angular velocity as a function of the ball position (right) for two illustrative participants (P2 upper panels, P10 lower panels). Shown are the trials from block 1 and block 12 for all three target size conditions (small: green; medium: blue and large: red). The thin gray lines in each panel represent the behavior observed over all cycles and the thicker black lines the cycle-averaged behavior. Two different movement strategies are discernable, which are relatively stable between target size conditions. Ball position is standardized to beam length (200 cm).

**Figure 5:**
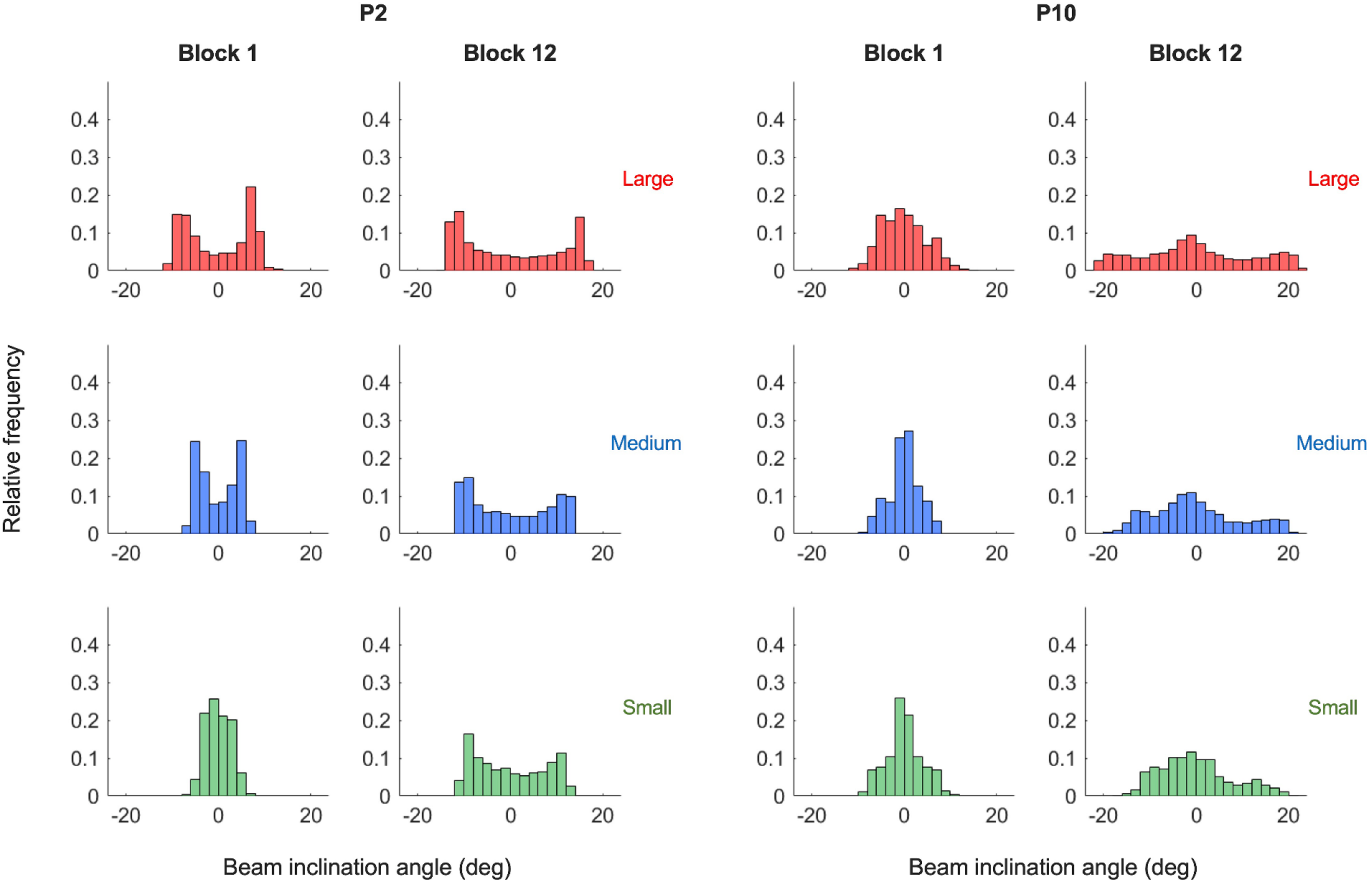
Beam inclination histograms of the two illustrative participants in (P2, left panels and P10, right panels) for blocks 1 and 12 under the three target size conditions (small: green; medium: blue and large: red). While some participants (left) spend much time at higher inclination angles, other participants (right) move more often to the middle.

To characterize the behavioral dynamics of the ball-and-beam system, we first addressed the dynamics of the ball on the beam. As illustrated in Figure 4 (left panels) by the phase portraits of ball motion for two characteristic participants P2 and P10 in practice blocks 1 and 12, the ball rolled continuously and in a close-to-harmonic manner in all trials and under each of the three target size conditions (see Supplementary Figure S1 for all participants). In the second place, we looked at the dynamics of the beam in relation to the ball. We found that the beam’s inclination angle oscillated in a manner that was characteristic for a participant. In order bring this out most clearly, we plotted the beam inclination velocity as a function of the ball position, thereby capturing the behavioral dynamics of this task in one graph. In the right panels of Figure 4 these ball-beam plots are shown for the same two characteristic participants (P2 and P10) in practice blocks 1 and 12 under all three target size conditions (See Supplementary Figure S1 for all participants). Two different beam-motion strategies arise from visual inspection of these graphs. The first strategy, adopted by the majority of the participants including P2 (Figure 4, upper right panel), was to maintain the beam at (close to) maximum inclination angle to set the ball into motion, before making one continuous movement to the opposite inclination peak in order to decelerate the ball and stop its motion within the target zone; it was then maintained there to set the ball in motion for the next half cycle. We refer to this strategy as the *extreme-stop* strategy. A second strategy, used by a minority of the participants including P10 (Figure 4, lower right panel), was to move the beam in two phases towards the opposite inclination, by stopping or slowing down the movement when the ball was about halfway between the targets. We call this strategy the *middle-stop strategy*. Although both strategies have in common that they are discrete as opposed to continuous, the locus at which participants stopped or slowed down their motion was different. This becomes even clearer when considering the histograms of the beam’s inclination angle, which captures the distribution of the inclination angle within a trial (Figure 5). Extreme stoppers exemplified by P2 (Figure 5, left) spend most time at maximum or minimum inclination angles (U-shaped histograms), while middle stoppers exemplified by P10 (Figure 5, right; see Supplementary Figure S2 for all participants) spend more time at inclination angle zero (bell-shaped histograms). Based on these results, we decided to use the shape ratio (SR), defined as the ratio between the average absolute inclination angle and half of the average peak-to-peak amplitude, to quantify the different strategies.

### 3.3 Beam motion

**Figure 6:**
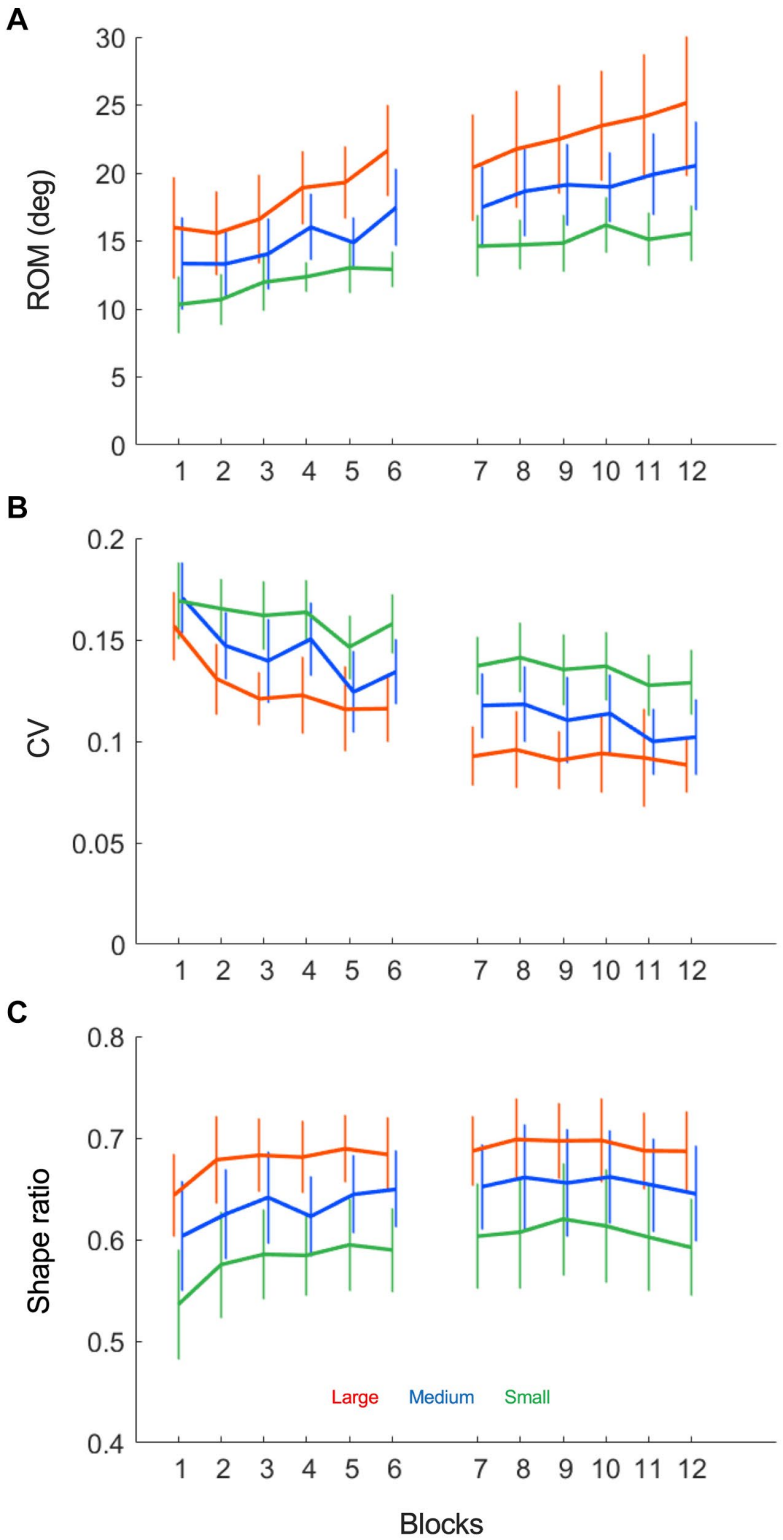
Beam motion variables as a function of practice blocks under each of the three target size conditions (small: green, medium: blue and large: red). **(A)** The average beam range of motion (ROM), defined as the average peak-to-peak amplitude in degrees, increases over practice in all conditions. **(B)** The average beam coefficient of variability (CV), defined as the average standard deviation divided by the range of motion, decreases over practice in all conditions. **(C)** The average beam shape ratio (SR), defined as the average absolute inclination angle divided by half the range of motion, is nearly invariant over practice, while different between task conditions.

Based on the above interpretation of the ball-beam phase planes, we had quantified three characteristic aspects of the beam motion, which we compared between conditions and over trials with a Condition (3) x Practice (12) repeated measures ANOVA (summarized in Table 1). First of all, the beam’s range of motion (ROM), defined as the average peak-to-peak amplitude in degrees, increased significantly over practice (Figure 6A, *F* (2.79, 41.82) = 14.94, *p* < 0.001, *η^2^_p_* = 0.50). The ROM was larger when the target sizes were larger (*F* (1.08, 16.24) = 28.79, *p* < 0.001, *η^2^_p_* = 0.66) and it also increased at a stronger rate in these conditions (*F* (22, 330) = 2.49, *p* < 0.001, *η^2^_p_* = 0.14). Secondly, the beam’s coefficient of variation (CV) declined significantly over trials (Figure 6B, *F* (4.91, 73.71) = 22.26, *p* < 0.001, *η^2^_p_* = 0.60), a finding that was visible in all target size conditions. Moreover, the CV was significantly different between conditions, such that the relative variability was higher when the target sizes were smaller (*F* (1.38, 20.62) = 43.15, *p* < 0.001, *η^2^_p_* = 0.74). Thirdly, the shape ratio (SR) showed an increasing, but non-significant trend (Figure 6C, *F* (4.44, 66.64) = 2.20, *p* = 0.072, *η^2^_p_* = 0.13). SR progressed slightly over the first few trials after which it stabilized for the rest of the two practice sessions (*F* (4.44, 66.64) = 2.20, *p* = 0.072, *η^2^_p_* = 0.13). However, a comparison of the task conditions revealed that the SR was different depending on the target size (*F* (1.08, 16.25) = 52.09, *p* < 0.001, *η^2^_p_* = 0.78). This is to say that the SR was larger with larger target sizes.

### 3.4 Relationship between ball-level and beam-level performance

To assess how the three beam motion variables related to the ball-level performance on this task, we computed Pearson correlation coefficients between each of the three beam motion variables and all three ball-related indicators of performance (target hits, ball speed and accuracy ratio). As shown in Table 2, the beam’s ROM correlated moderately high with the number of target hits in all conditions, indicating that a higher movement amplitude usually resulted in a larger number of target hits and was thus associated with task success. The ROM was also strongly correlated with the ball speed in all task conditions, while correlations of the ROM with the accuracy ratio of the ball were low and non-significant, indicating that the range of motion had impacted the ball’s speed but not its accuracy. The beam’s coefficient of variation, on the other hand, was negatively correlated with the number of target hits when targets were large in size, but uncorrelated in the smallest target size condition. This suggested that good performances were generally associated with low amounts of beam motion variability in the larger target size conditions, but not in the smallest target size conditions. The correlations of the beam’s CV with the ball speed and accuracy ratio were low and non-significant in all task conditions. Lastly, the SR of the beam’s movement was uncorrelated with the number of target hits in all three target size conditions, suggesting that task performance was independent from the shape of the beam movement. The SR only had a significant and moderately negative correlation with the ball’s accuracy ratio in the small target size condition. This indicated that an extreme-stop strategy was associated with lower a ratio of points over cycles in the small target size conditions, but not in the other two task conditions.

**Table 2:** Pearson correlation coefficients between the three beam motion variables (beam ROM, beam CV and beam SR) and each of the three indicators of task performance target hits, ball speed and accuracy ratio in each of the three target size conditions (small, medium, large).

	Target Size					
	Small		Medium		Large	
	r (14)	*p*	r (14)	*p*	r (14)	*p*
Target hits
Beam ROM	+0.72	0.002	+0.62	0.011	+0.63	0.009
Beam CV	−0.55	0.026	−0.46	0.076	−0.09	0.752
Beam SR	−0.20	0.466	−0.11	0.695	−0.22	0.408
Ball speed						
Beam ROM	+0.97	< 0.001	+0.94	< 0.001	+0.84	< 0.001
Beam CV	−0.32	0.231	−0.30	0.265	−0.35	0.183
Beam SR	−0.41	0.115	−0.09	0.732	−0.40	0.128
Accuracy ratio						
Beam ROM	−0.25	0.355	−0.20	0.461	+0.15	0.581
Beam CV	−0.27	0.302	−0.11	0.664	+0.22	0.409
Beam SR	+0.16	0.551	−0.14	0.609	−0.59	0.002

## 4 Discussion

In this study we investigated individual skill acquisition in the solo setting of the manual ball-and-beam paradigm, a challenging task that requires a continuous perceptuomotor coordination to control a ball rolling on a hand-held beam. In particular, we were interested in the behavioral dynamics (Warren, 2006) that would arise from learning a perception-action coupling in this task. Based on research on reciprocal aiming tasks with similar instructions—i.e., moving an end-effector as fast and accurately as possible between two targets– we hypothesized that the emergent behavioral dynamics would be dependent on the accuracy constraints of the task. More specifically, we expected that small targets would invoke a discrete strategy of moving the beam, while large targets would lead to a more continuous movement strategy. To test this hypothesis, we had participants practice the task in two individual training sessions with three different target size conditions.

To start with, practice on the task resulted in an overall improvement of the performance (see Figure 2), most clearly demonstrated by the increase in the number of target hits per trial. This finding was in accordance with our earlier study using this task (Hafkamp et al., 2023) and provided an indication that participants acquired (the fundamentals of) the skill to control the motion of the ball on the beam. Also in line with Hafkamp et al. (2023), we found that the increase in the number of target hits was accompanied by a strong (35%) increase in the average ball speed from block 1 to 12 and a smaller (12%) increase in the ball’s accuracy (hits/cycles). In combination, these results demonstrated that participants learned to control the ball’s motion at increasingly high speeds, suggesting that they indeed acquired an improved and task-specific coupling of perception and action.

New in this contribution was the introduction of three different target sizes, which were used to investigate the influence of task-level accuracy demands on the performance. As predicted by the speed-accuracy trade off in perceptuomotor control (Fitts, 1954; Fitts and Peterson, 1964; Plamondon and Alimi, 1997), we found that larger target sizes resulted in higher ball speeds (Figure 2B) and lower ball motion consistencies around the targets zones (Figure 2D). In other words, the lower the demand on accuracy, the higher the speed of the rolling ball. Our goal in this contribution, however, was to look beyond the outcome measures of ball speed and accuracy and to study the underlying behavioral dynamics that gave rise to these movement properties. To do so, we did not limit our analysis to the dynamics of the ball alone, but combined it with a dynamical analysis of the concurrent and underlying movements of the beam, since the two are relatedly through a gravity-mediated coupling. In accordance with our expectations, the inertial properties of the ball resulted in a continuous ball motion in almost all blocks of practice. Only in the smallest target size conditions did the ball occasionally (though very rarely) come to a full stop, breaking the otherwise rhythmic motion of the ball between the two targets. Thus, whereas the speed-accuracy trade-off in manual reciprocal aiming tasks usually arises from significant qualitative changes in the dynamics of the end-effector (Mottet and Bootsma, 1999; Mottet et al., 2001; Bongers et al., 2009; Huys et al., 2010), the changes in the dynamics of a ball rolling on a beam are more subtle and appear to be of a quantitative rather than of a qualitative nature (Figure 4, left panels). This is to say that ball showed a similar limit-cycle dynamics in all target size conditions. On the one hand, this might indicate that no qualitative behavioral changes occurred in response to the accuracy demands on the task. On the other hand, the physical properties of the system might have concealed any behavioral changes, meaning that differences in the beam’s motion patterns were not visible in the ball’s motion on the beam. To further investigate this, we interpreted the dynamics of the beam’s inclination angle in relation to the ball.

The cross-correlation analysis of the ball position with the beam’s inclination angle (Figure 3) revealed that a maximum correlation between the time series was found at small positive time-lags, indicating that the motion of the beam lagged slightly behind that of the rolling ball, for at least the largest part of the average movement cycle on a trial. Over the course of a trial, participants thus systematically adapted their beam motion to the (perceived) ball motion. In contrast to the ball motion, however, the pattern of movement of the beam’s inclination angle was not harmonic or continuous, but mostly discrete (Figure 4, right panels). Following the literature on reciprocal aiming (Mottet and Bootsma, 1999; Mottet et al., 2001; Bootsma et al., 2004; Bongers et al., 2009; Huys et al., 2010), we had hypothesized that large accuracy demands would give rise to a discrete movement strategy of the beam, while low demands on accuracy would lead to a more continuous movement of the beam. In the present study we did not find such a qualitative contrast between target size conditions in the nature of the end-effector (here beam) dynamics. All participants adopted a discrete pattern of beam motion in all task conditions, independent of the demands on accuracy. Thus, continuous sinusoidal movements of the beam were never adopted as a fruitful task solution by the participants, even when target sizes were large. This suggests that the type of indirect control characterizing the ball-and-beam task may be fundamentally different from the direct control of the end-effector in manual reciprocal aiming and therefore must be interpreted in a different way. Further indication for this was provided by the remarkable finding of stable inter-individual differences in the beam dynamics deployed. This was in contrast with the behavioral dynamics as found in traditional paradigms of reciprocal aiming, which are generally comparable between individuals (Mottet and Bootsma, 1999; Mottet et al., 2001; Bongers et al., 2009; Huys et al., 2010). The individual differences could not be defined on a continuum from discrete to continuous strategies, but were instead defined by the *locus* of stopping or slowing down within the beam’s cycle of motion (see Figure 5).

The first strategy that we found was referred to as an *extreme-stop strategy.* In this scenario, adopted by the majority of the participants, the beam motion was paused at the minimum/maximum inclination angle to set the ball into motion. After this pause, participants produced one, continuous movement to the other extreme (i.e., from minimum to maximum inclination or vice versa) to decelerate the ball and stop it in the next target zone; participants then maintained the beam at this extreme position so as to repeat the process for accelerating the ball in the other direction. In a second scenario, participants did not produce one, continuous movement between extremes, but divided each half cycle into two phases. In the first phase, (almost) immediately after onset of the ball motion, the beam was brought to inclination angle zero, while in the second phase the beam moved from zero to the opposite extreme angle to decelerate the ball and stop it in the next target zone. These two phases were thus separated by a short stopping or slowing down of the beam movement, leading us to refer to this beam-motion pattern as a *middle-stop strategy*. Both strategies shared a discrete rather than continuous character, which contributed to the small delay of the beam inclination relative to the ball position that was found in the cross-correlation analysis. Nevertheless, the locus of stopping or slowing down differed between the two strategies, which had significant implications for the control process underlying the performance. To understand the significance of this difference, we need to consider the gravitational effect of stopping at these two different loci in the beam’s cycle.

At the extreme points of inclination, the driving (tangential) component of the gravitational force acting on the ball is at its peak. This implies that the ball accelerates/decelerates maximally over the period that the beam motion is stopped at these loci. Any small difference in the beam’s inclination angle at this point therefore has a considerable impact on the ball’s motion, which is to say that the required timing and extent of the subsequent (sub) movement is highly constrained by this inclination angle. At inclination angle zero, however, the ball rolls without gravity-induced acceleration. As such, the ball is no longer increasing speed and the constraints on the timing and extent of the subsequent movement are thus lower than at the extremes. This is reflected in the larger coefficient of variation of the beam movement of middle-stoppers (P10: *M* ± *SD*: 0.14 ± 0.02)than of extreme-stoppers (P2: *M*  ± *SD*: 0.10 ± 0.02). To acquire a high average ball speed on a trial and score many points, the middle-stopper is forced to produce large inclination angles to compensate for the relatively long time that is spent around inclination angle zero. Yet, one could also turn this argument around. To prevent the ball from rolling too fast, participants might have had to compensate for large inclination angles by slowing down the beam around inclination angle zero, leading to a middle-stop strategy. Either way, the two-phased beam dynamics constituted a significantly different approach to the perceptuomotor problem posed by the ball-and-beam system than the one-phased dynamics. In other words, it reflected a qualitatively different intrinsic pattern of coordination on the task, a different ‘preferred’ behavioral organization.

In the framework of coordination dynamics, the process of learning such stable patterns of coordination has also been conceptualized as the convergence onto certain attractors in the solution space of a task (Schöner et al., 1992; Zanone and Kelso, 1992; Kostrubiec et al., 2012). Intriguingly, both of the two reported strategies in the ball-and-beam task allowed for the achievement of task success, since the adopted strategy was not significantly correlated with the performance measures of target hits, ball speed or ball accuracy (except for the smallest target size condition, see below for a possible explanation for this). Thus, multiple loci in the solution space could function as an attractor in the task performance. Although this does not explain why individuals converged onto one rather than the other solution, it may explain why the intrinsic differences between individuals were stable and invariant over practice: Since neither of the two strategies appeared to be better than the other, the drive for participants to transition to another pattern, either abruptly or through a gradual change of location in the solution space (Jacobs and Michaels, 2007; Pacheco et al., 2019), was absent. In this respect, the task differed fundamentally from traditional rhythmic task paradigms as used in the domain of coordination dynamics, in which often only one pattern of coordination is the optimally stable (e.g., the in-phase pattern of coordination for rhythmic interlimb movements, Haken et al., 1985). It is also in contrast with other complex and continuous perceptuomotor tasks, in which also only one optimal strategy usually emerges from practice (e.g., pedalo task, Chen et al., 2005; ski-simulation task Vereijken et al., 1992, 1997). We note that over extended and intensive practice on the ball-and-beam task, the lower energetic efficiency of the middle-stop strategy might eventually bring participants to adopt the more efficient extreme-stop strategy. This, however, remains but speculative for the time being.

If participants did not change their movement strategy to adapt to the different target size conditions, then the question arises as to what caused the speed-accuracy trade-off at the level of the rolling ball. First of all, we found that the range of motion (ROM) of the beam varied in accordance with the accuracy demands, such that smaller target sizes gave rise to smaller ROMs of the inclination angle, regardless of the adopted strategy (Figure 6). The beam’s ROM was strongly correlated with the ball speed, implying that participants used it directly to modulate the speed of the ball (see also Hafkamp et al., 2023). Moreover, the ROM was highly correlated with the number of target hits—in particular in the large target condition—suggesting that the ROM was a strong indicator for task success when the accuracy demands were low. Secondly, the relative variability of the beam motion (CV) was significantly larger when the target size was smaller, even though its correlation with the number of target hits was low in these conditions. A possible reason for this apparent contradiction could be that the larger variability of the beam was caused by a lack of control of the ball for some participants, but for others gave rise to an increased use of corrective sub-movements to enhance control under the high accuracy constraints. This hypothesis is supported by the finding that in small target size conditions, a middle-stop strategy was associated with a higher accuracy of the ball motion, while an extreme stop strategy led to a lower ball accuracy ratio. The use of sub-movements has been found as a task solution in goal-directed pointing tasks as well (e.g., Keele and Posner, 1968; Meyer et al., 1990, for a review: Plamondon and Alimi, 1997) and it might reflect a general strategy to preserve accuracy in a goal-directed movement. This conclusion should be drawn with caution here, however, as the indirect form of control operating in this ball-and-beam paradigm may be fundamentally different from more direct forms of control, such as in manual aiming tasks.

The beam’s range of motion and variability also played a crucial role in the performance progression over blocks. The process of skill acquisition was characterized by two distinct phases of learning. In the first phase, participants explored the ball-beam relationship to establish a general movement strategy. This exploratory phase was accompanied by a decrease in variability and an increase in the correlation between the ball position and beam inclination (Figure 3C). In the literature, increases in the correlation between action-related and perception-related variables have been associated with improvements of the perceptuomotor coupling on a task (e.g., Jacobs et al., 2012). After this exploratory stage, the correlation between ball and beam motions stabilized and the established movement strategy—either an extreme-stop or a middle-stop dynamics—no longer changed over the duration of practice. To further improve performance in the second stage of learning, participants gradually increased the beam’s range of motion, while decreasing its relative movement variability (Figure 6). In other words, they performed with more risk and higher consistency. Although this process of refinement was most clearly visible for the large target sizes, it took place in all task conditions. Such a two-staged process of skill acquisition is characteristic of *de novo* learning and has been found in many other perceptuomotor tasks before (Newell and McDonald, 1994; for a review see Guimarães et al., 2020). In studies using a cyclical ski-simulation task, for instance, Vereijken et al. (1997) demonstrated that in a first exploratory stage participants moved the platform from left to right with a very high variability and a low range of motion, while participants found a stable and consistent movement pattern with a larger range of motion in a second stage of learning. Similar to the findings of the present study, they reported that participants gradually increased their range of motion to improve performance in this latter stage of learning.

We conclude that individual skill acquisition in the solo setting of the manual ball-and-beam task is characterized by a gradual refinement of one of two alternative movement strategies that emerges in an early stage of learning. The chosen strategy can best be defined by the locus of stopping or slowing down in the beam’s movement cycle and reflects the behavioral dynamics that arise from the perceptuomotor coupling on the task. In other words, the movement strategy is an expression of how each player coordinates the cyclical motion of the beam with the continuous motion of the ball rolling on that beam. These results are particularly interesting for our broader purpose of understanding interpersonal coordination through a joint action version of the ball-and-beam task. In joint action, both individuals not only have to coordinate their movements with the rolling ball on the beam, but also with the concurrent actions of another individual. Given the stable differences in the behavioral dynamics between individuals, we expect that dyadic performances are shaped by the idiosyncrasies of the individuals that constitute the dyads. In other words, based on the present results we expect that the adopted strategy (either extreme-stop or middle-stop) will be among the pertinent characteristics that are transferred from the solo to the joint action setting of the ball-and-beam task. This would imply that who interacts with whom may be relevant to the form as well as the stability of the interpersonal coordination. In future work we will investigate this interaction by combining the currently presented insights into individual skill acquisition with the study of joint action performances on the ball-and-beam task.

## Data Availability

The raw data supporting the conclusions of this article will be made available by the authors, without undue reservation.

## Footnotes

^1^ https://www.kinovea.org/
