# Supplementary material for: Perceptuomotor skill acquisition in a solo manual ball-and-beam task with varying accuracy requirements
Source: Front Psychol. 2024 Aug 29;15:1436099. doi: 10.3389/fpsyg.2024.1436099 (PMC11391423; doi:10.3389/fpsyg.2024.1436099)
Supplement: Supplementary file 1 [file Data_Sheet_1.pdf]

Supplementary Figure 1

Perceptuomotor skill acquisition in a solo manual ball-and-beam task with varying accuracy requirements

Marijn S. J. Hafkamp, Remy Casanova, Reinoud J. Bootsma*

Supplementary Figure 1. Plots of the ball velocity as a function of the ball position (left) and the beam angular velocity as a function of the ball position (right) for all 16 participants (from P1 to P16, one participant per page) for the trials from block 1 and block 12 under all three target width conditions (small: green; medium: blue and large: red). The thin gray lines in each panel represent the behavior observed over all cycles and the thicker black lines the cycle-averaged behavior. Ball position is standardized to beam length (196 cm).

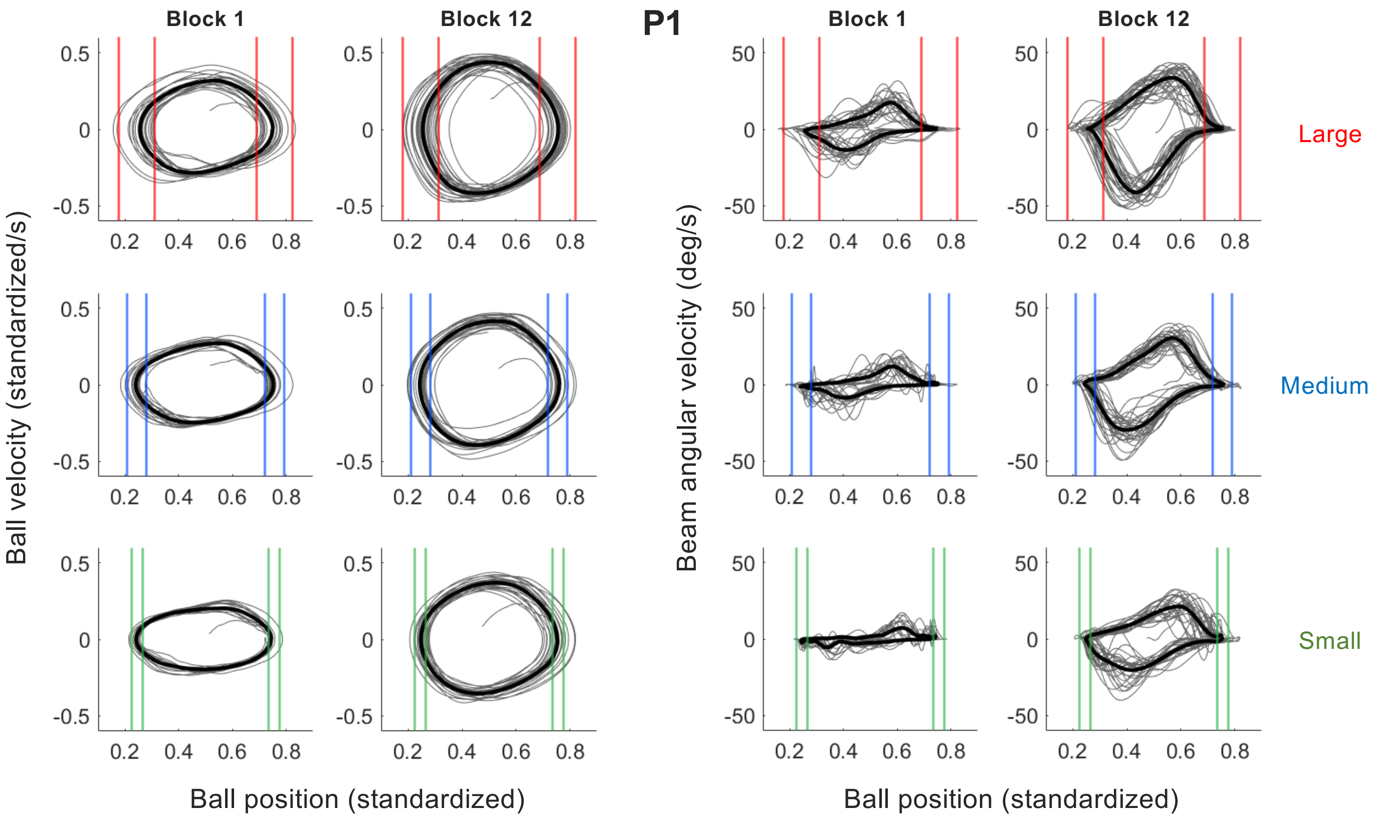
P1
Block 1
Block 12
Block 1
Block 12
Ball velocity (standardized/s)
Beam angular velocity (deg/s)
Ball position (standardized)
Ball position (standardized)
Large
Medium
Small

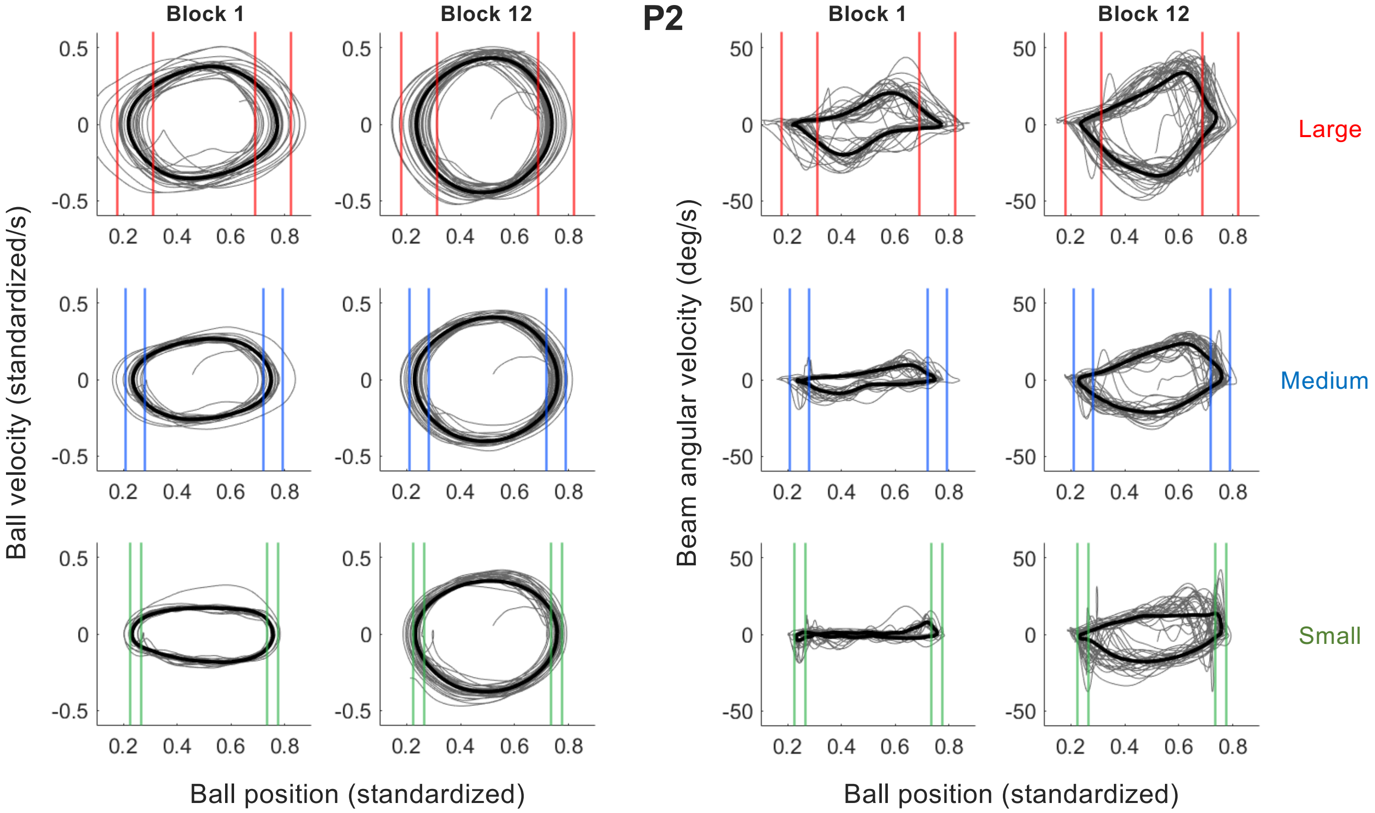
P2
Block 1
Block 12
Block 1
Block 12
Ball velocity (standardized/s)
Beam angular velocity (deg/s)
Large
Medium
Small
Ball position (standardized)
Ball position (standardized)

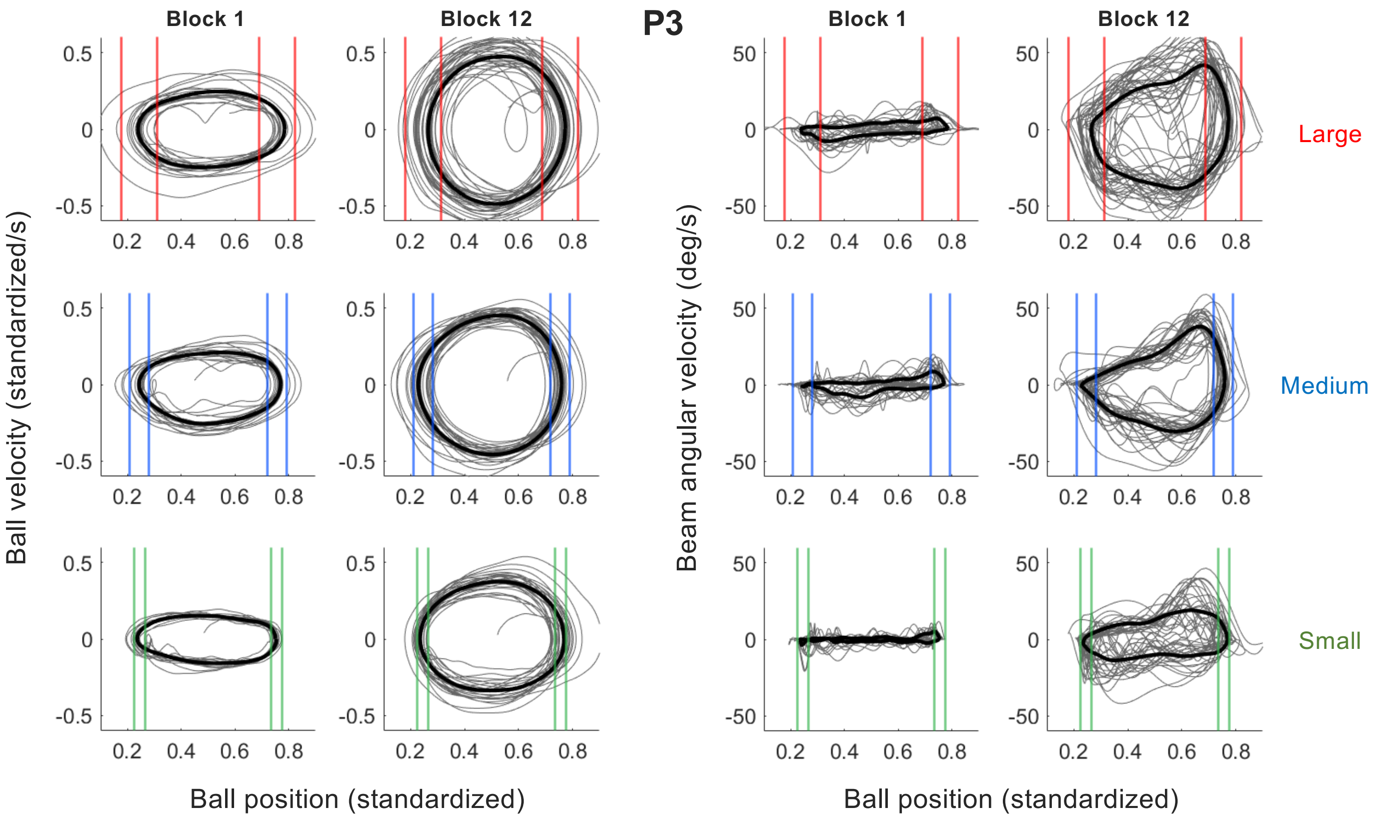
P3
Block 1
Block 12
Block 1
Block 12
Ball velocity (standardized/s)
Beam angular velocity (deg/s)
Ball position (standardized)
Ball position (standardized)
Large
Medium
Small
0.5
0
-0.5
50
0
-50
0.2
0.4
0.6
0.8

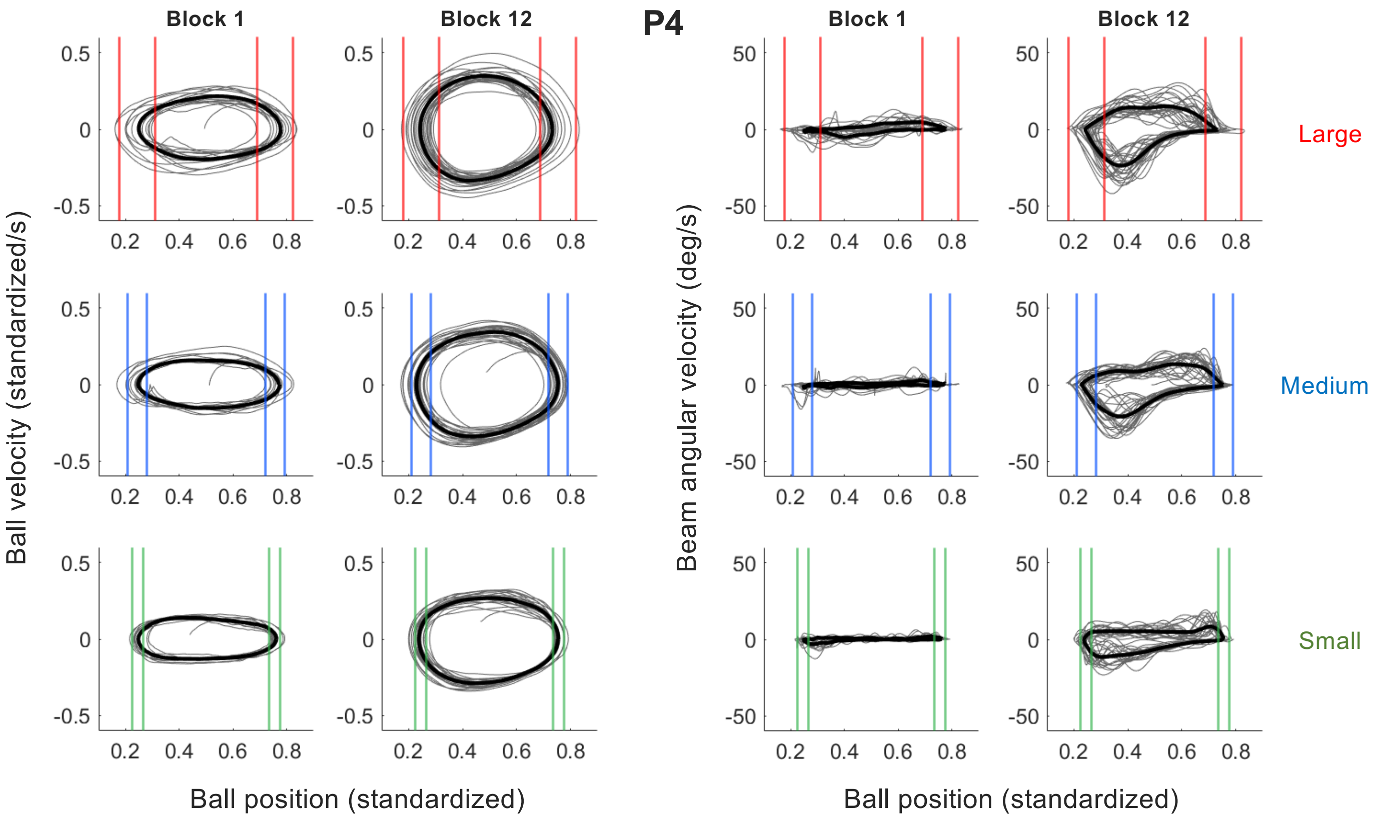
Block 1
Block 12
P4
Block 1
Block 12
Large
Medium
Small
Ball velocity (standardized/s)
Beam angular velocity (deg/s)
Ball position (standardized)
Ball position (standardized)

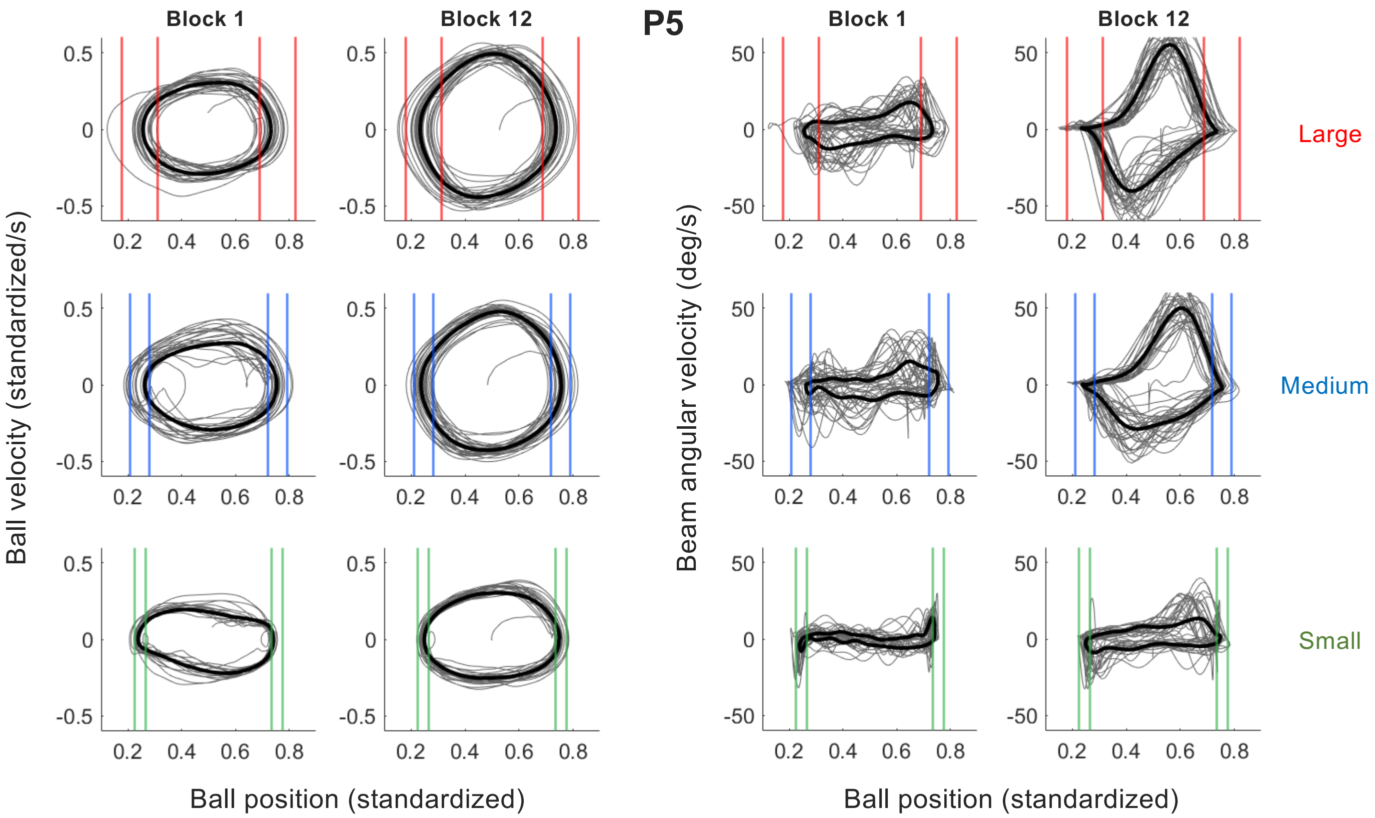

P5
Block 1
Block 12
Block 1
Block 12
Ball velocity (standardized/s)
Beam angular velocity (deg/s)
Ball position (standardized)
Ball position (standardized)
Large
Medium
Small

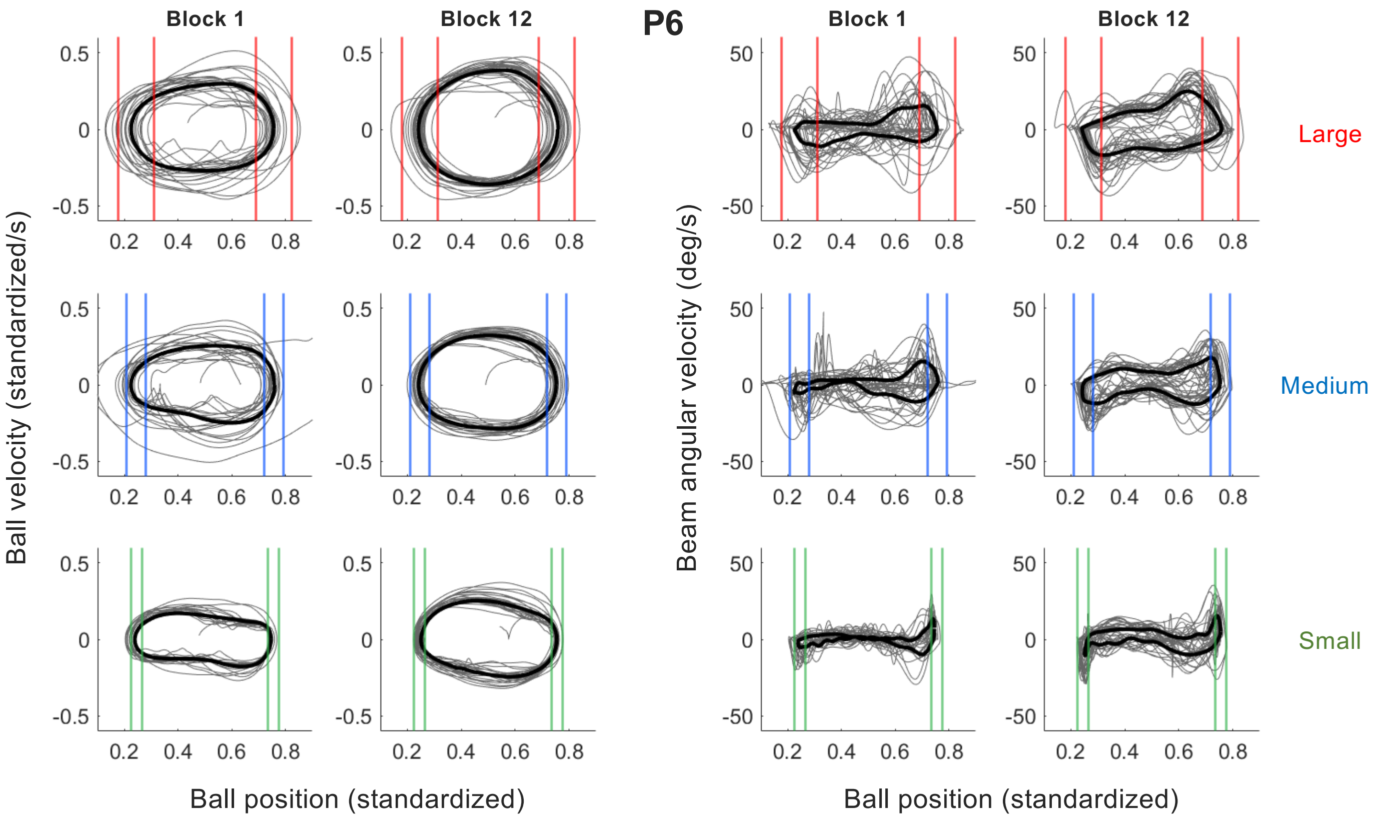
P6
Block 1
Block 12
Block 1
Block 12
Ball velocity (standardized/s)
Beam angular velocity (deg/s)
Ball position (standardized)
Ball position (standardized)
Large
Medium
Small
0.5
0
-0.5
50
0
-50
0.2
0.4
0.6
0.8

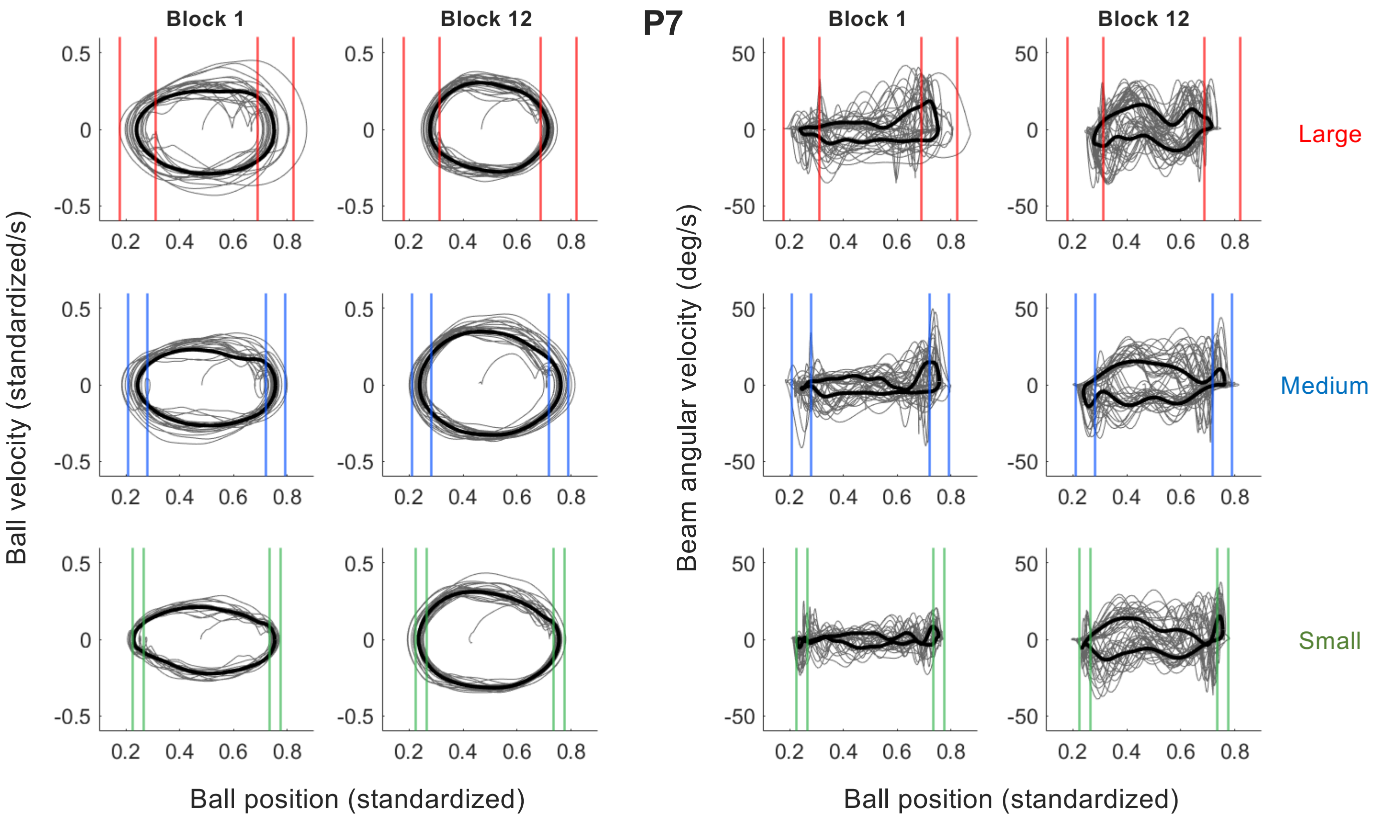
P7
Block 1
Block 12
Block 1
Block 12
Ball velocity (standardized/s)
Beam angular velocity (deg/s)
Ball position (standardized)
Ball position (standardized)
Large
Medium
Small

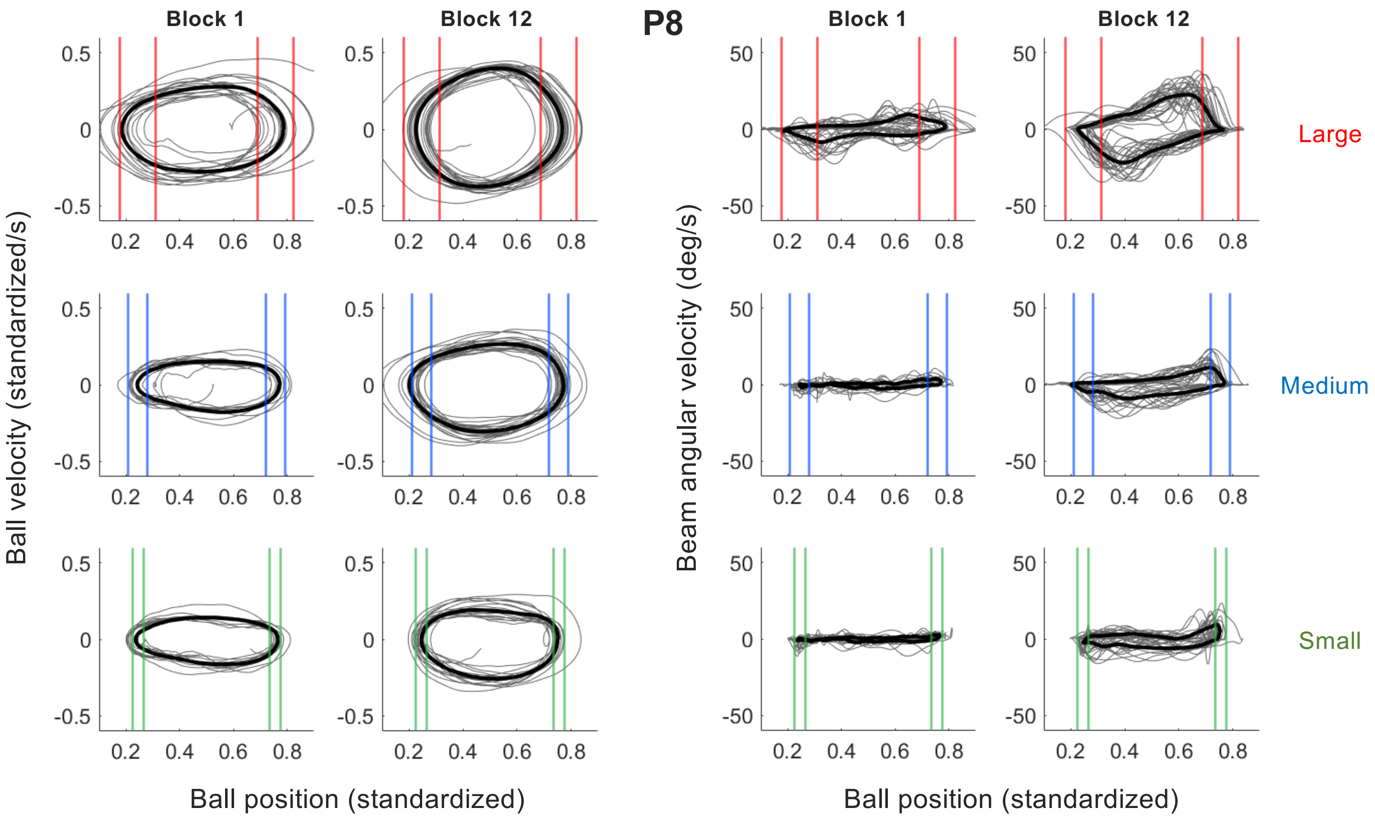
P8
Block 1
Block 12
Block 1
Block 12
Ball velocity (standardized/s)
Beam angular velocity (deg/s)
Large
Medium
Small
Ball position (standardized)
Ball position (standardized)

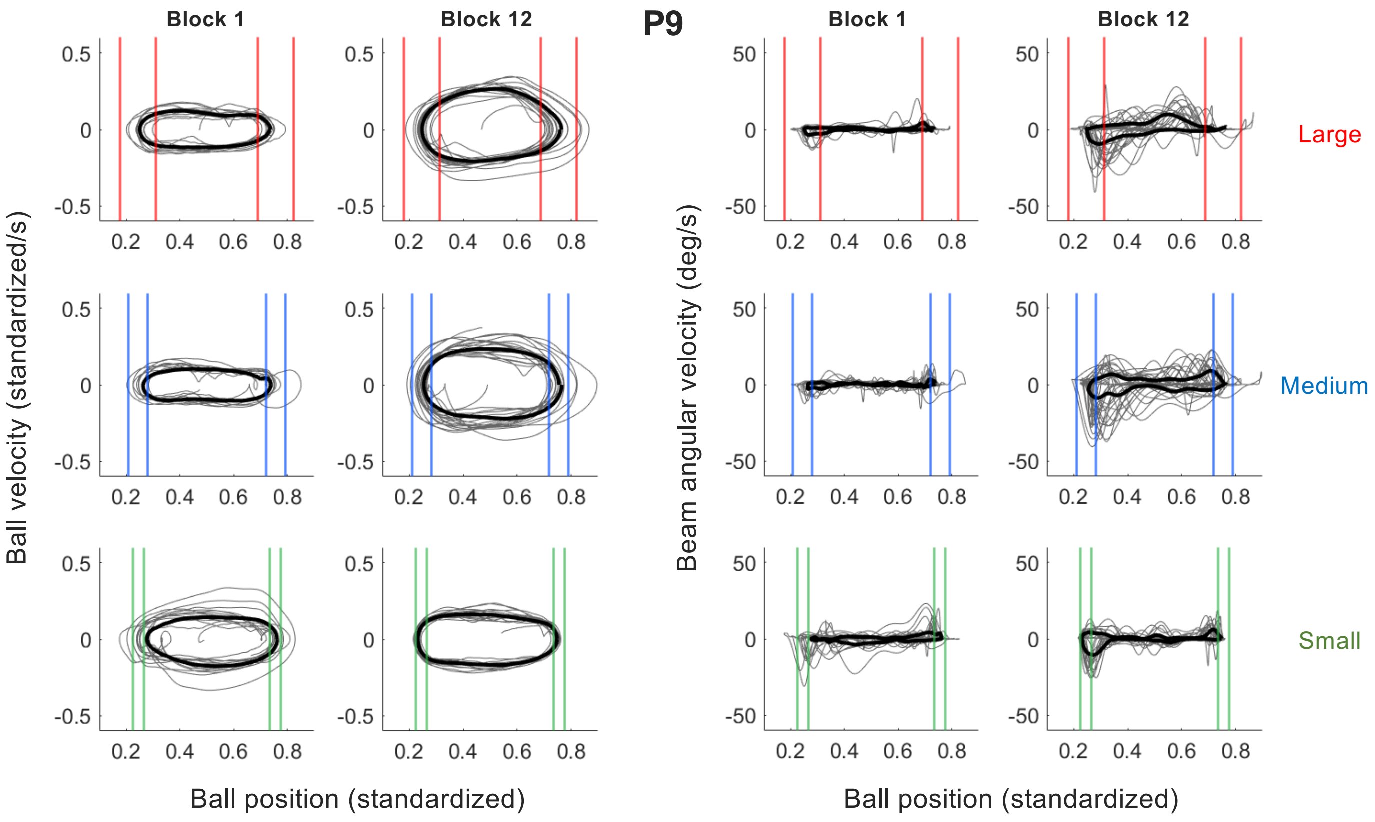
P9
Block 1
Block 12
Block 1
Block 12
Ball velocity (standardized/s)
Beam angular velocity (deg/s)
Large
Medium
Small
Ball position (standardized)
Ball position (standardized)

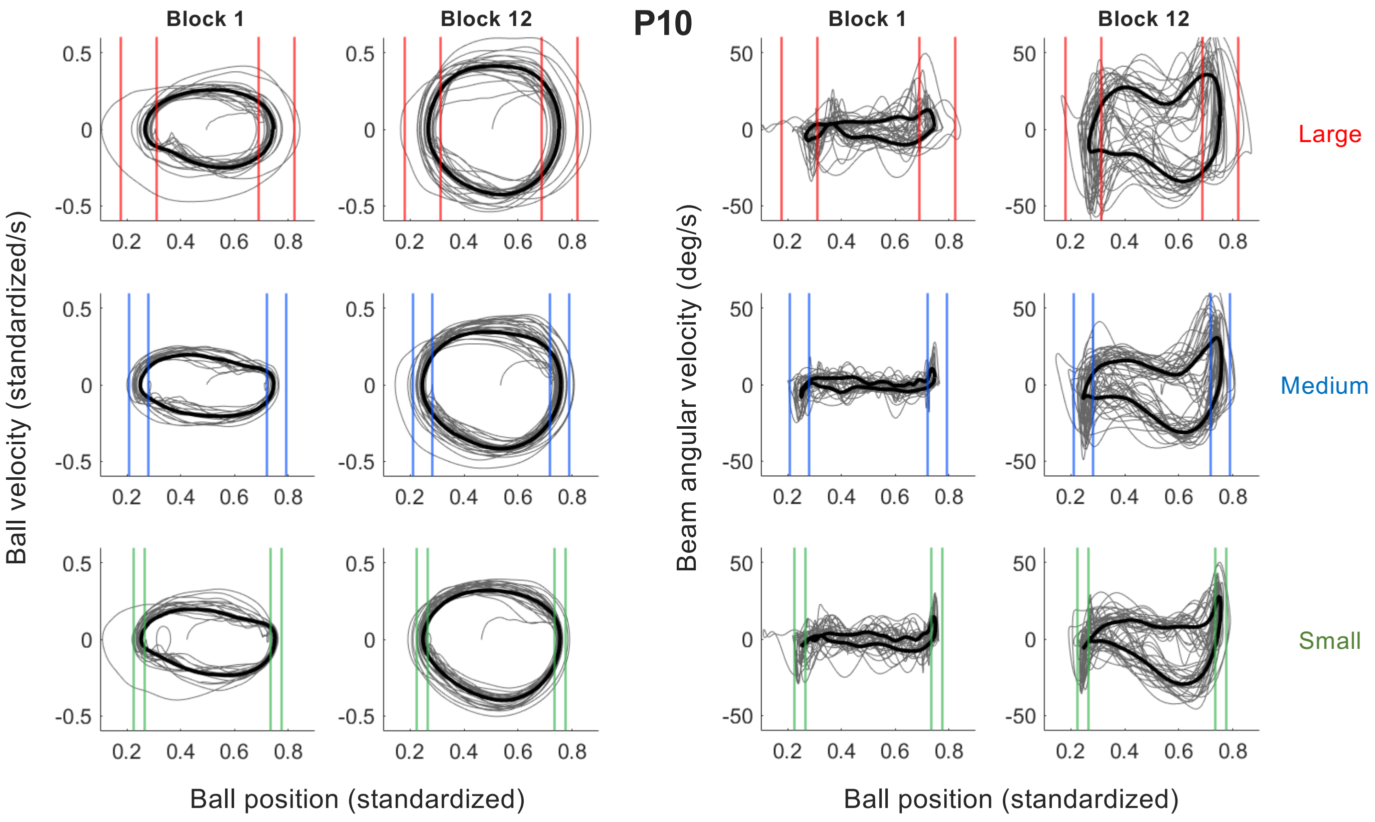
P10
Block 1
Block 12
Block 1
Block 12
Ball velocity (standardized/s)
Beam angular velocity (deg/s)
Ball position (standardized)
Ball position (standardized)
Large
Medium
Small

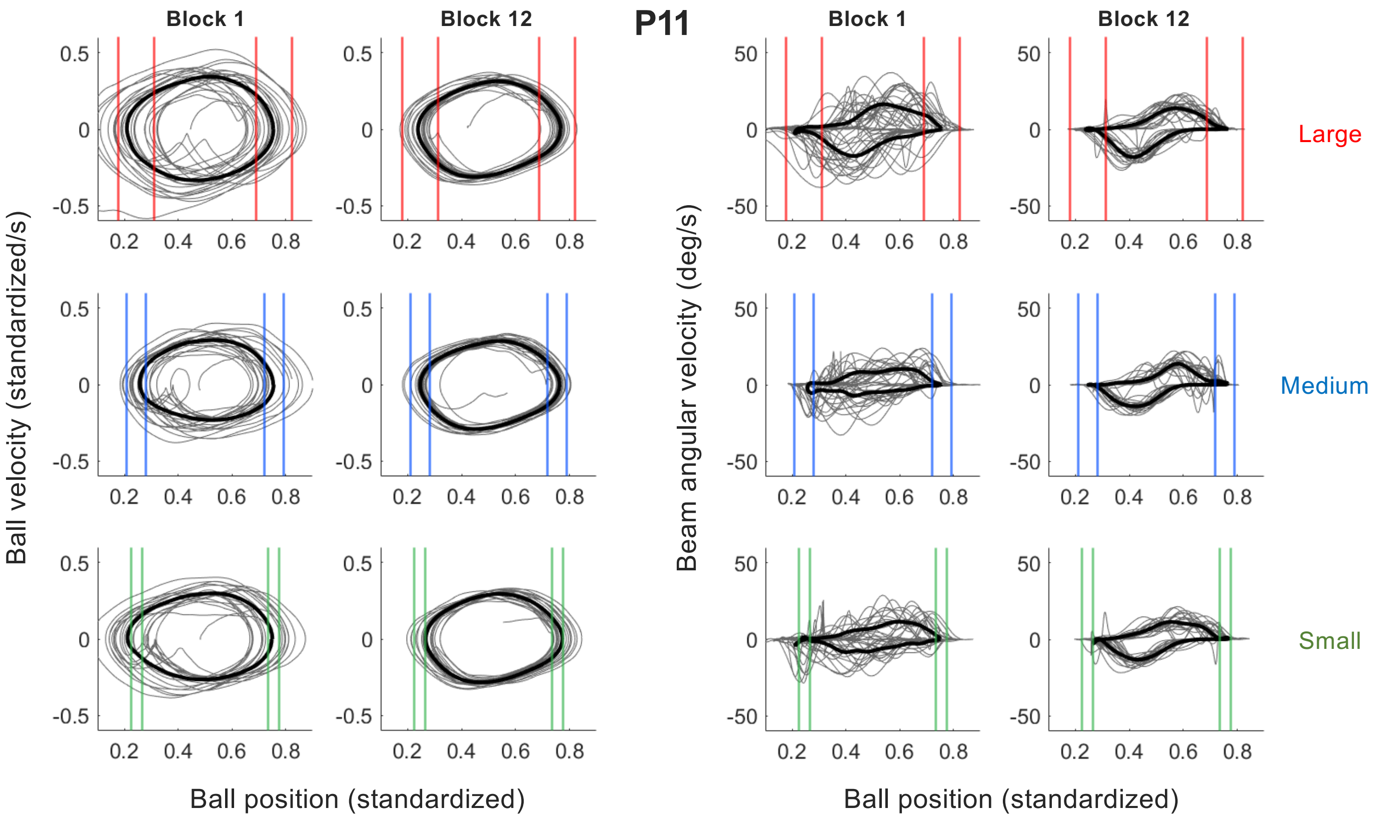
P11
Block 1
Block 12
Block 1
Block 12
Ball velocity (standardized/s)
Beam angular velocity (deg/s)
Ball position (standardized)
Ball position (standardized)
Large
Medium
Small

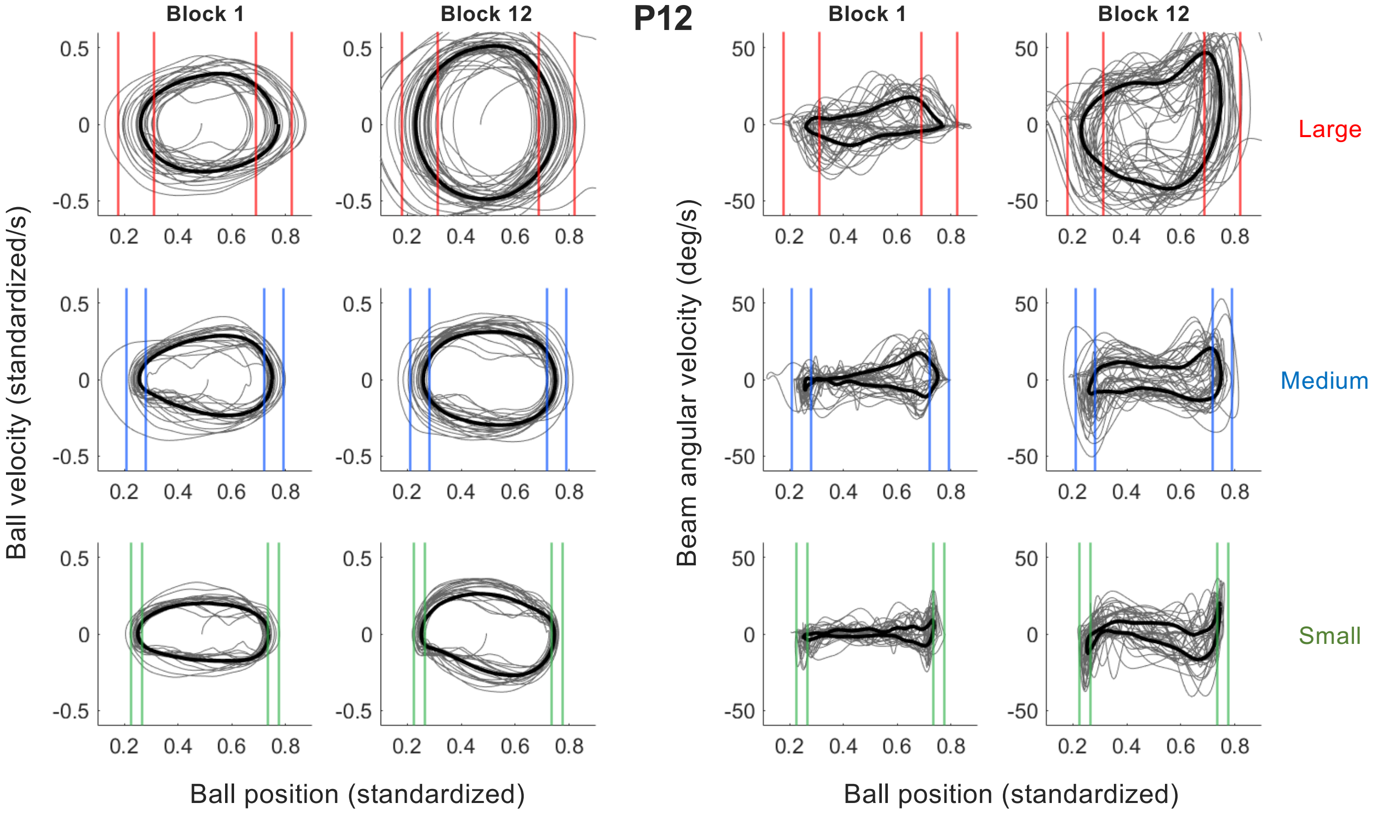
P12
Block 1
Block 12
Block 1
Block 12
Ball velocity (standardized/s)
Beam angular velocity (deg/s)
Ball position (standardized)
Ball position (standardized)
Large
Medium
Small

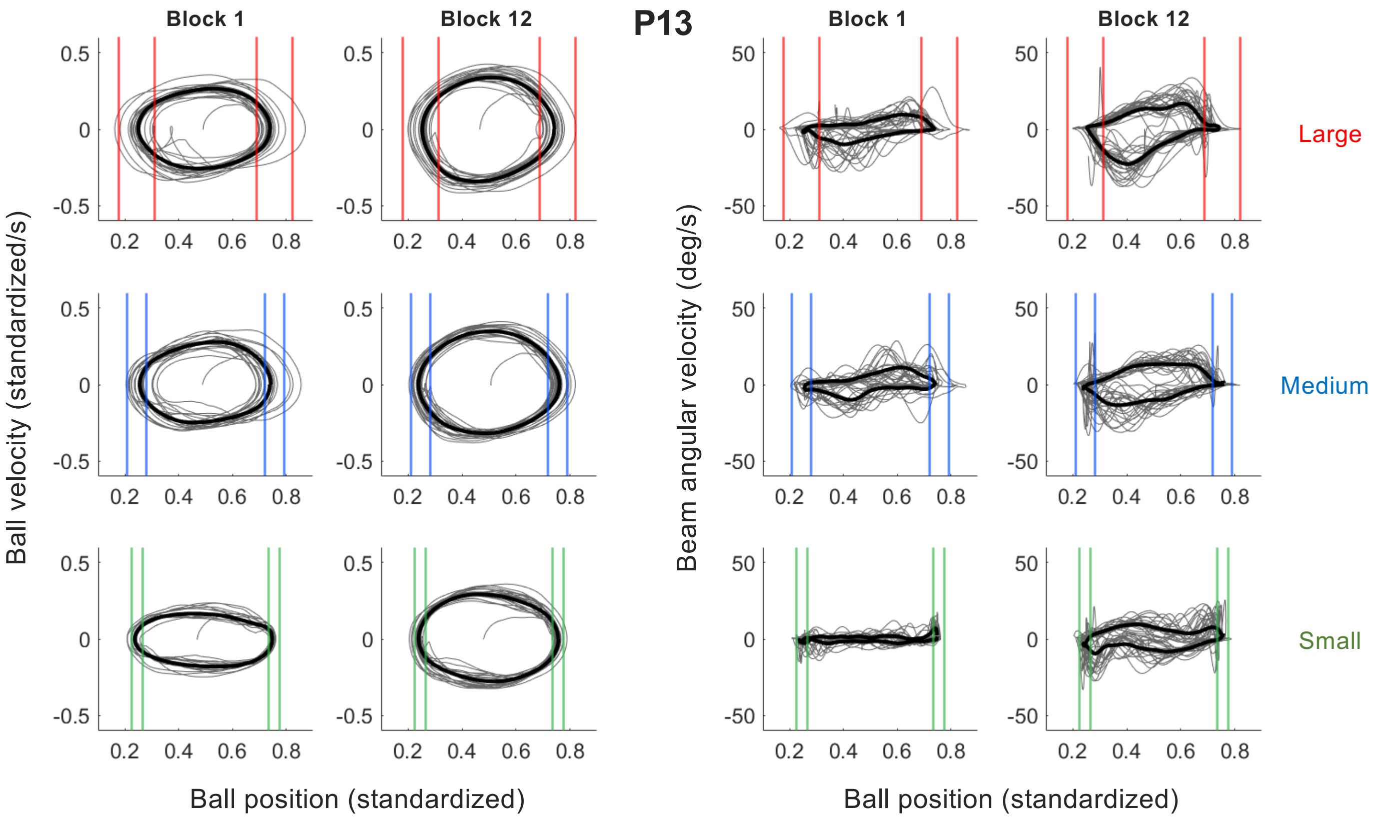
P13
Block 1
Block 12
Block 1
Block 12
Ball velocity (standardized/s)
Beam angular velocity (deg/s)
Ball position (standardized)
Ball position (standardized)
Large
Medium
Small
0.5
0
-0.5
50
0
-50
0.2
0.4
0.6
0.8

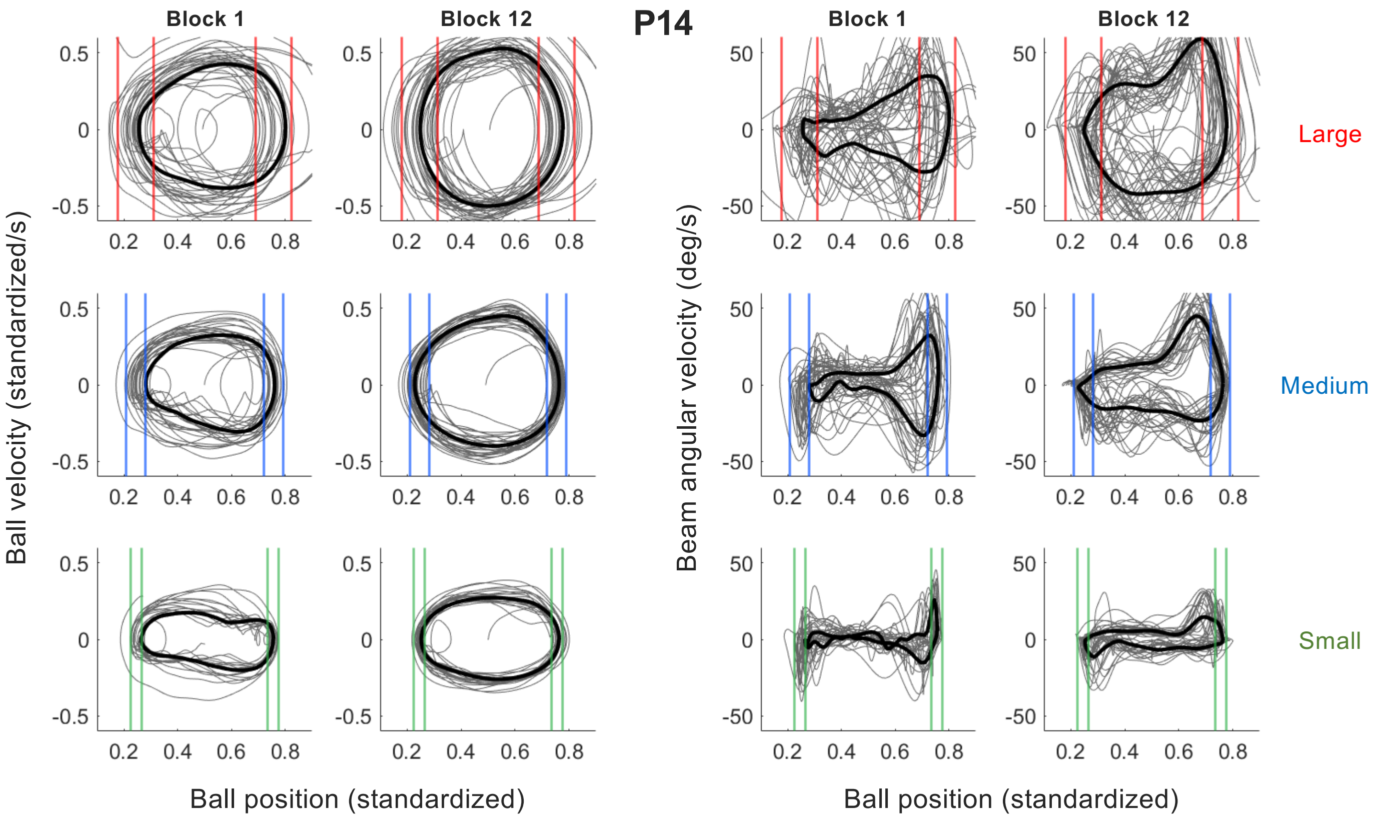
P14
Block 1
Block 12
Block 1
Block 12
Ball velocity (standardized/s)
Beam angular velocity (deg/s)
Ball position (standardized)
Ball position (standardized)
Large
Medium
Small
0.5
0
-0.5
50
0
-50
0.2
0.4
0.6
0.8

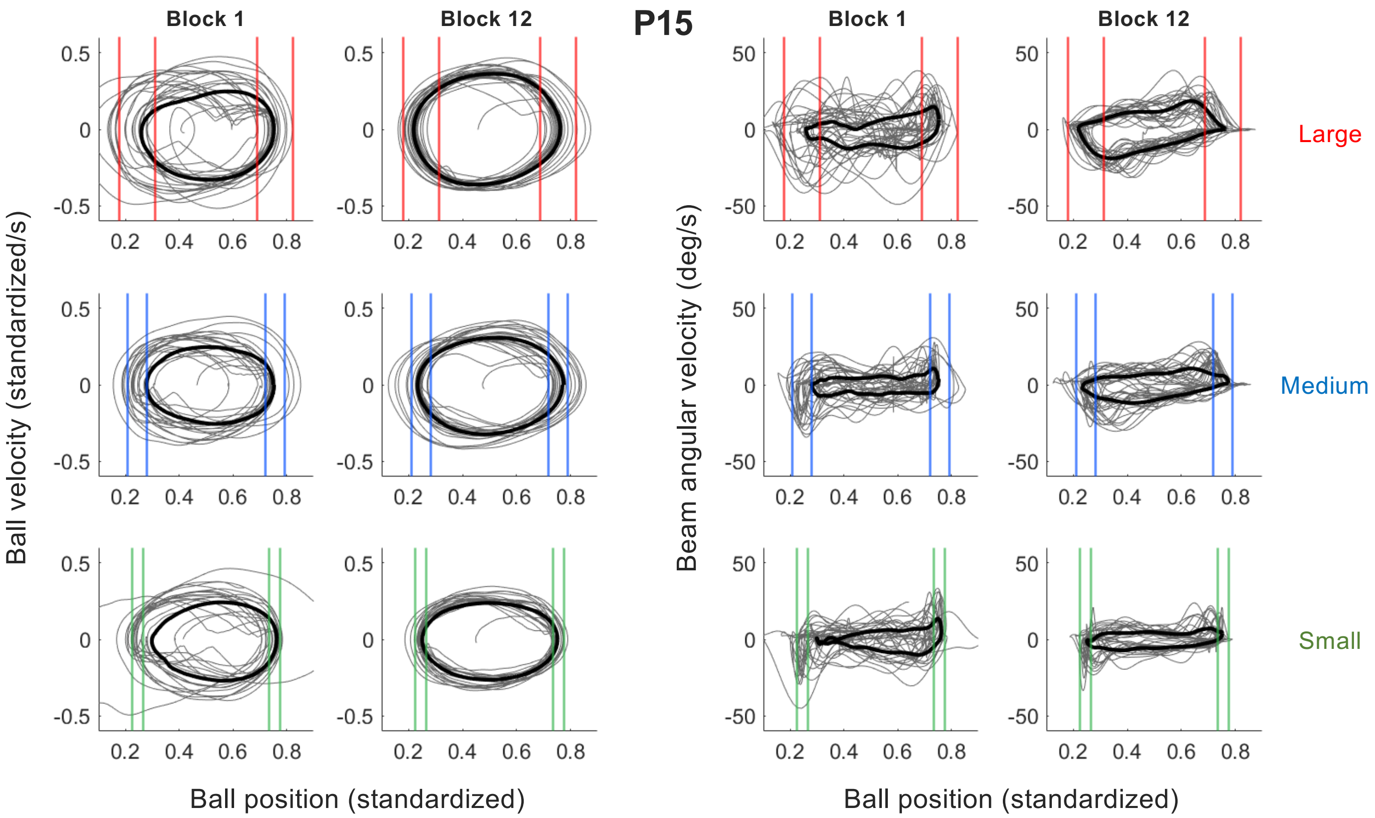

P15
Block 1
Block 12
Block 1
Block 12
Ball velocity (standardized/s)
Beam angular velocity (deg/s)
Ball position (standardized)
Ball position (standardized)
Large
Medium
Small

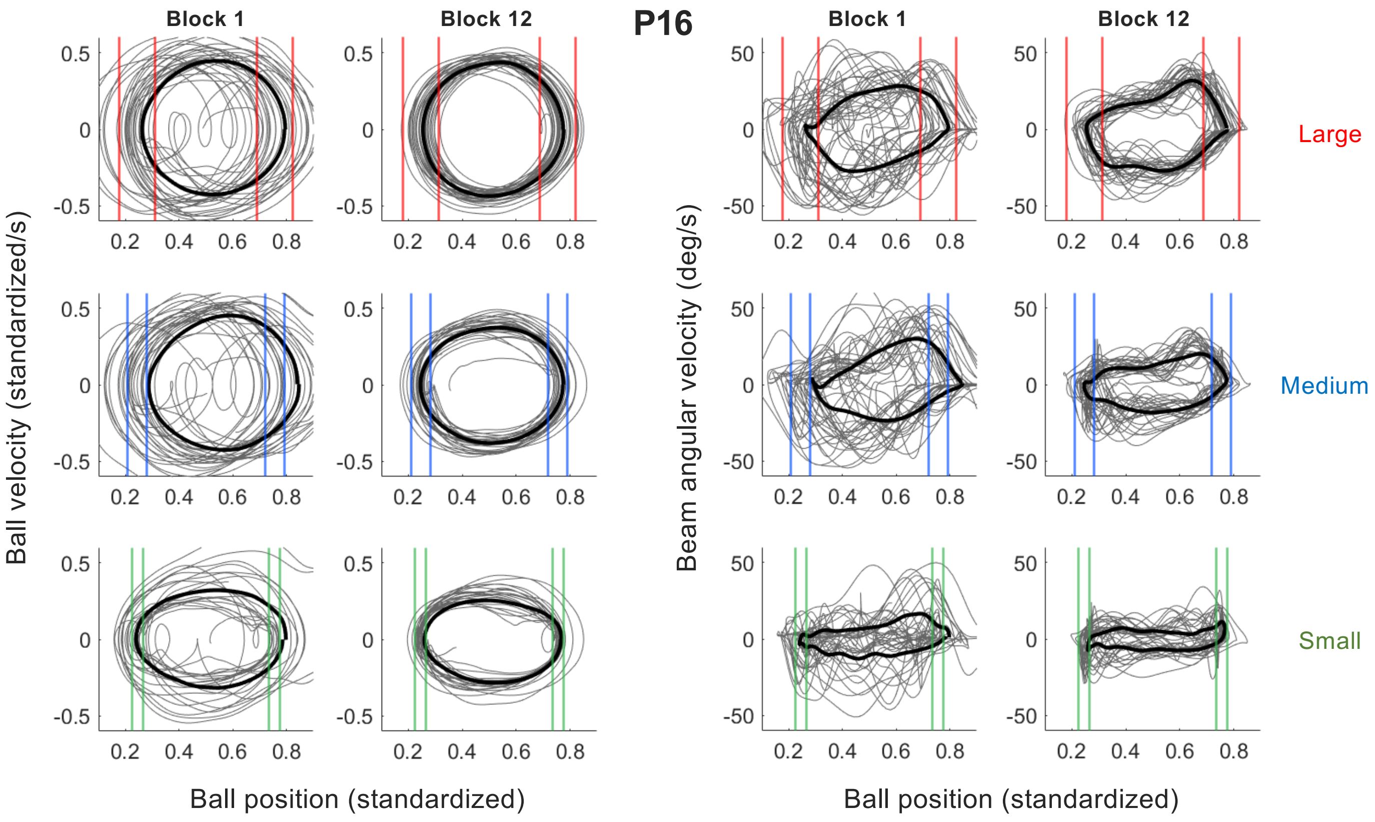
P16
Block 1
Block 12
Block 1
Block 12
Ball velocity (standardized/s)
Beam angular velocity (deg/s)
Large
Medium
Small
Ball position (standardized)
Ball position (standardized)
