# Supplementary material for: Perceptuomotor skill acquisition in a solo manual ball-and-beam task with varying accuracy requirements
Source: Front Psychol. 2024 Aug 29;15:1436099. doi: 10.3389/fpsyg.2024.1436099 (PMC11391423; doi:10.3389/fpsyg.2024.1436099)
Supplement: Supplementary file 2 [file Data_Sheet_2.pdf]

Supplementary Figure 2

Perceptuomotor skill acquisition in a solo manual ball-and-beam task with varying accuracy requirements

Marijn S. J. Hafkamp, Remy Casanova, Reinoud J. Bootsma*

Supplementary Figure 2. Beam inclination histograms of all participants (from P1 to P16, one participant per page) for trials of blocks 1 and 12 under the three target width conditions (small: green; medium: blue and large: red).

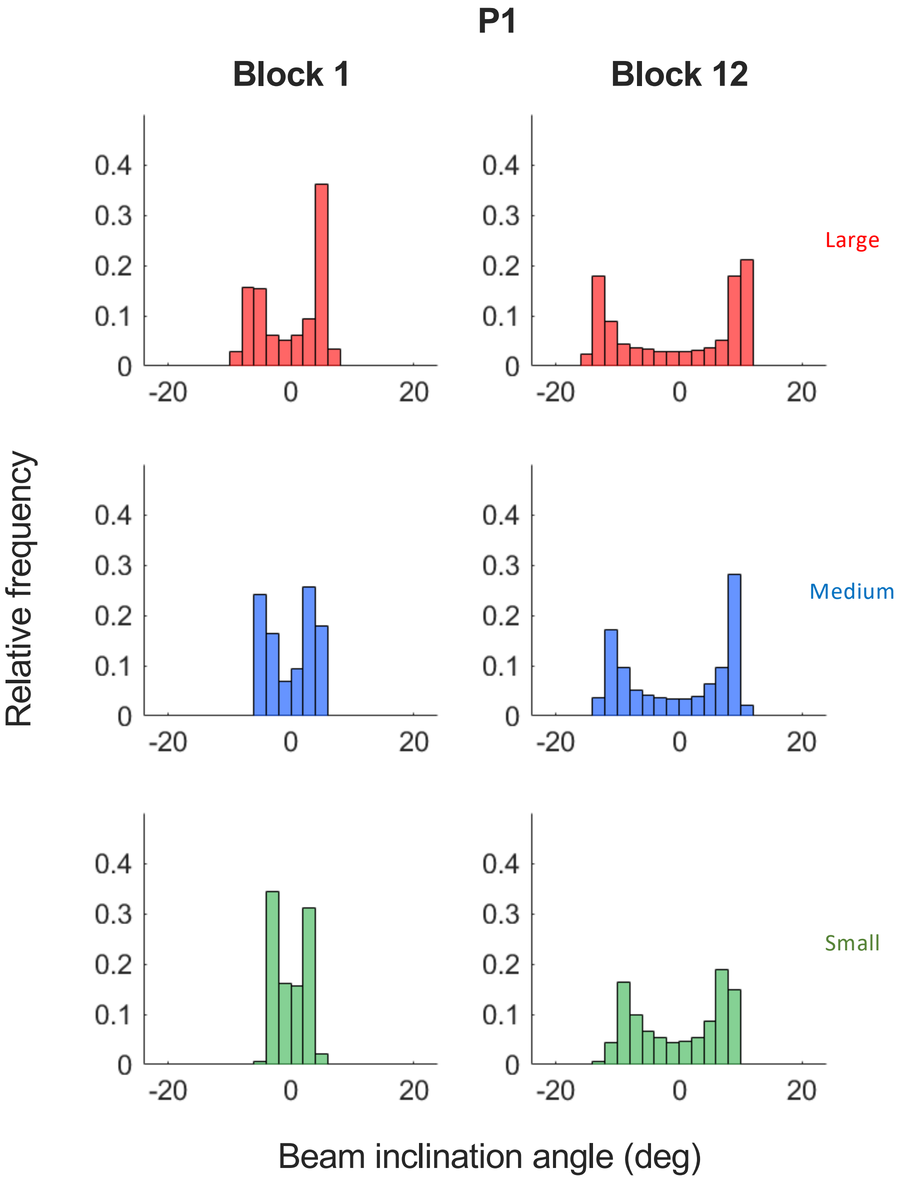
P1
Block 1
Block 12
Large
Medium
Small
Relative frequency
Beam inclination angle (deg)
0
0.1
0.2
0.3
0.4
-20
0
20

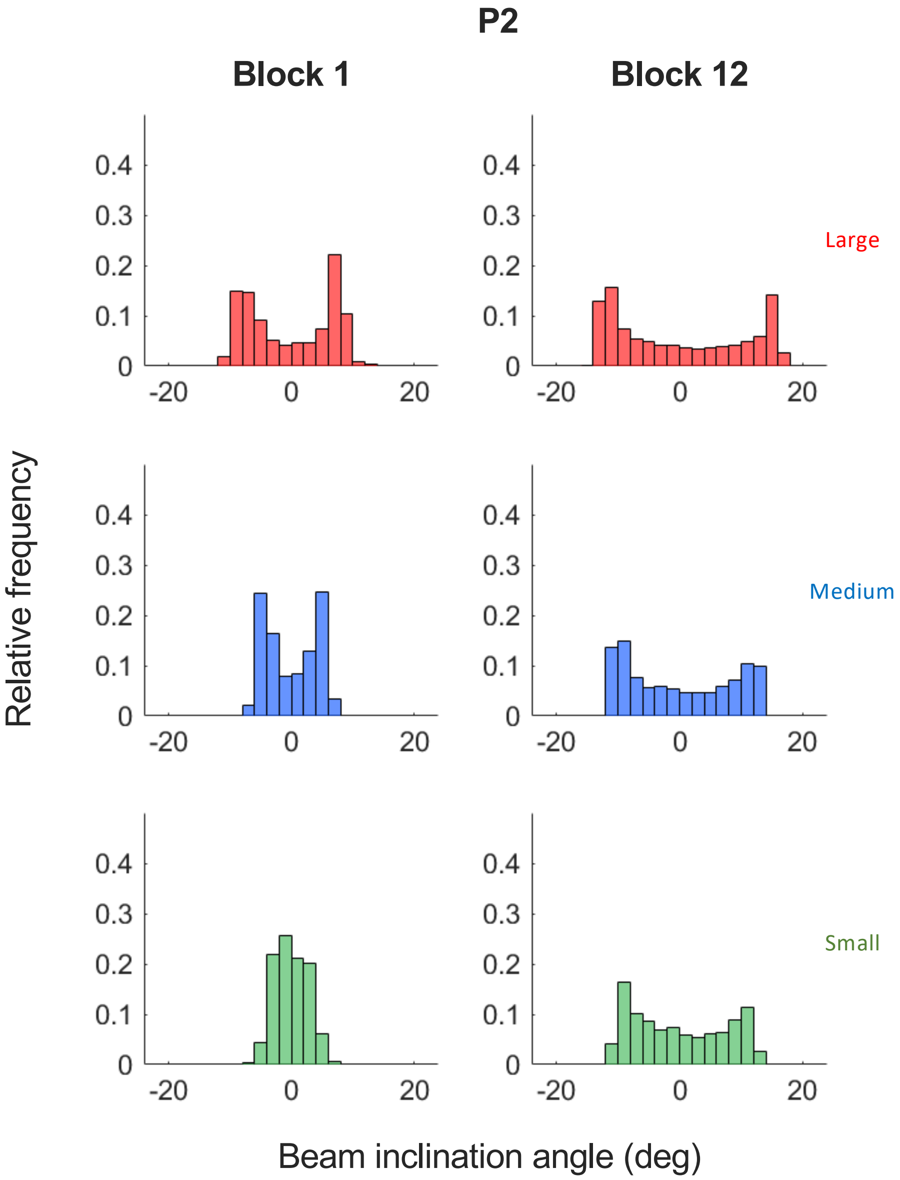
P2
Block 1
Block 12
Large
Medium
Small
Relative frequency
Beam inclination angle (deg)
0.4
0.3
0.2
0.1
0
-20
0
20

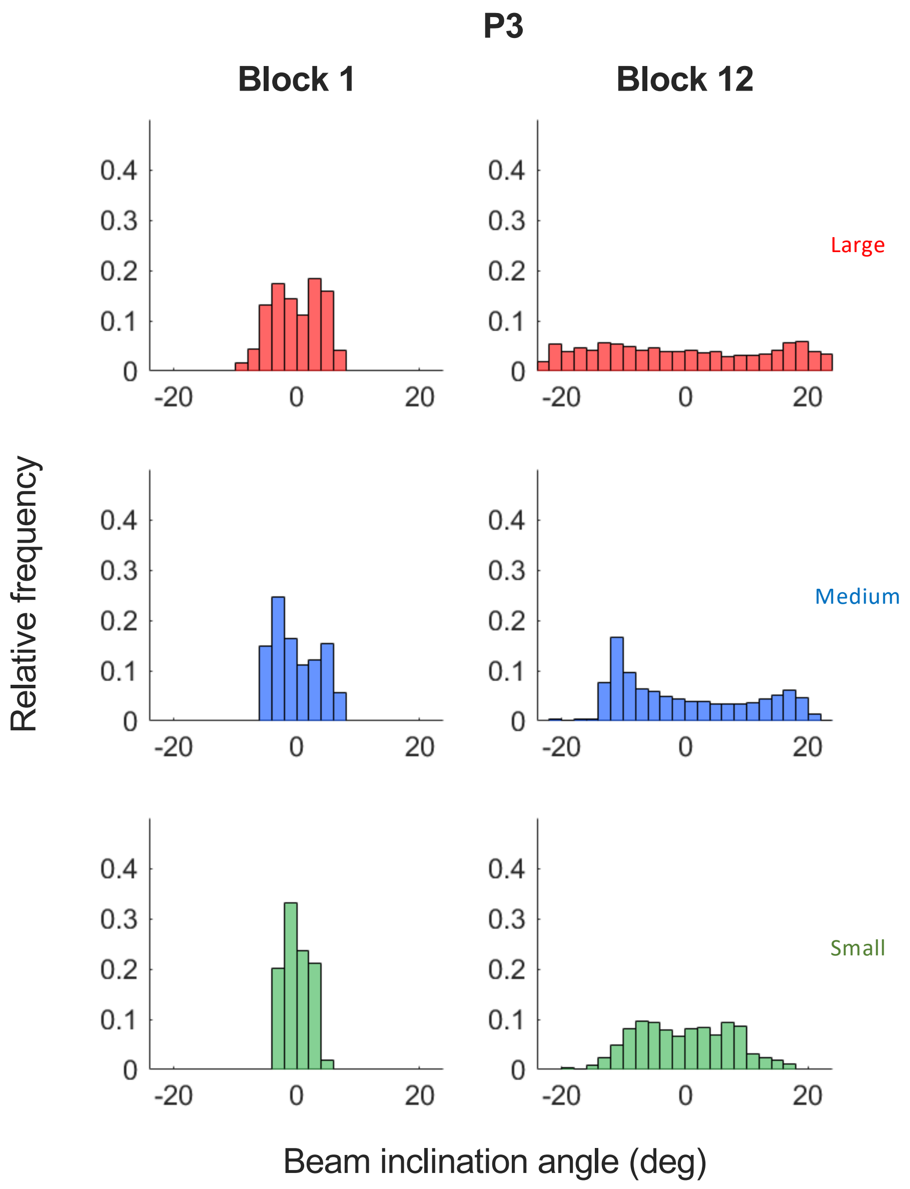
P3
Block 1
Block 12
Large
Medium
Small
Relative frequency
Beam inclination angle (deg)
0.4
0.3
0.2
0.1
0
-20
0
20

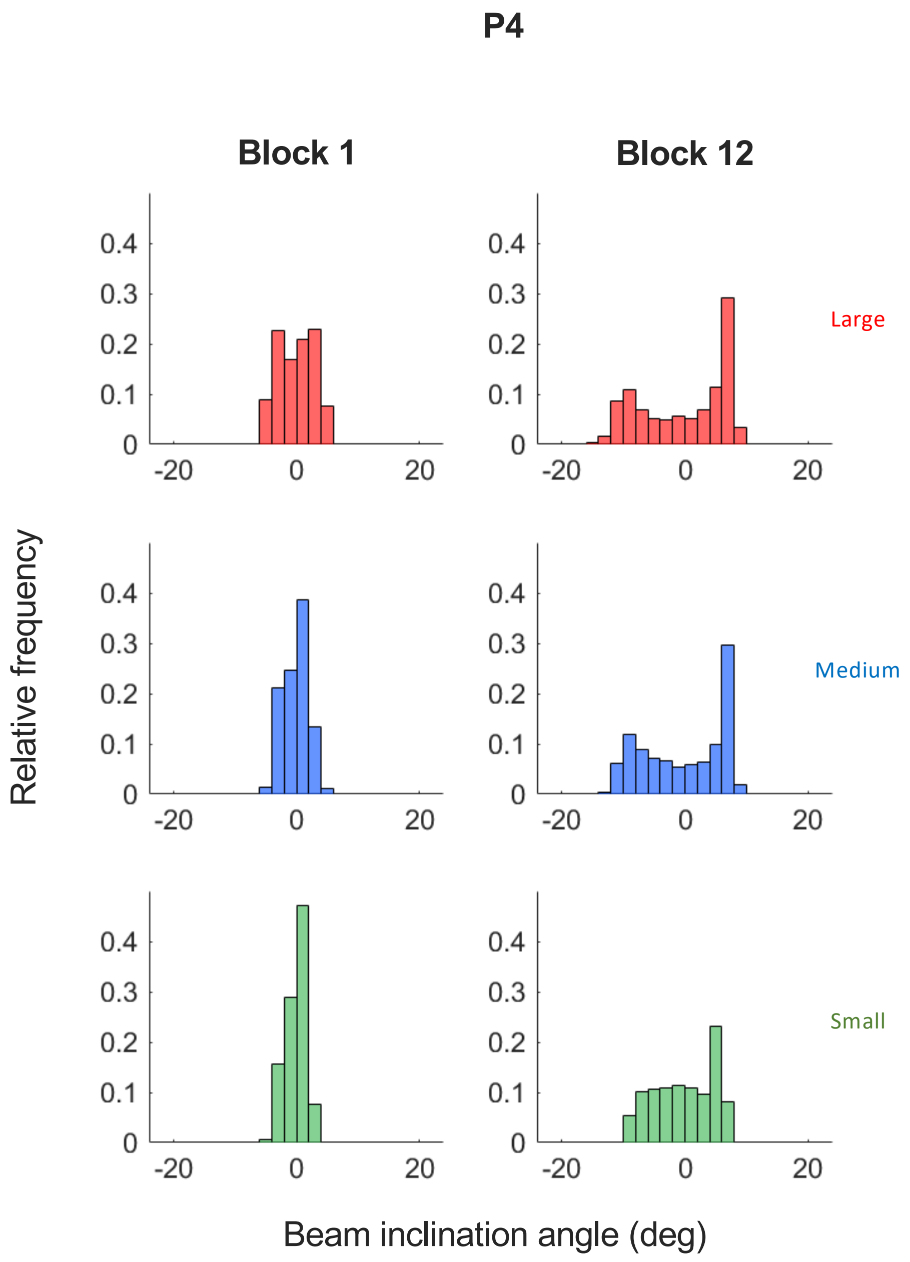
P4
Block 1
Block 12
Relative frequency
Large
Medium
Small
0.4
0.3
0.2
0.1
0
-20
0
20
Beam inclination angle (deg)

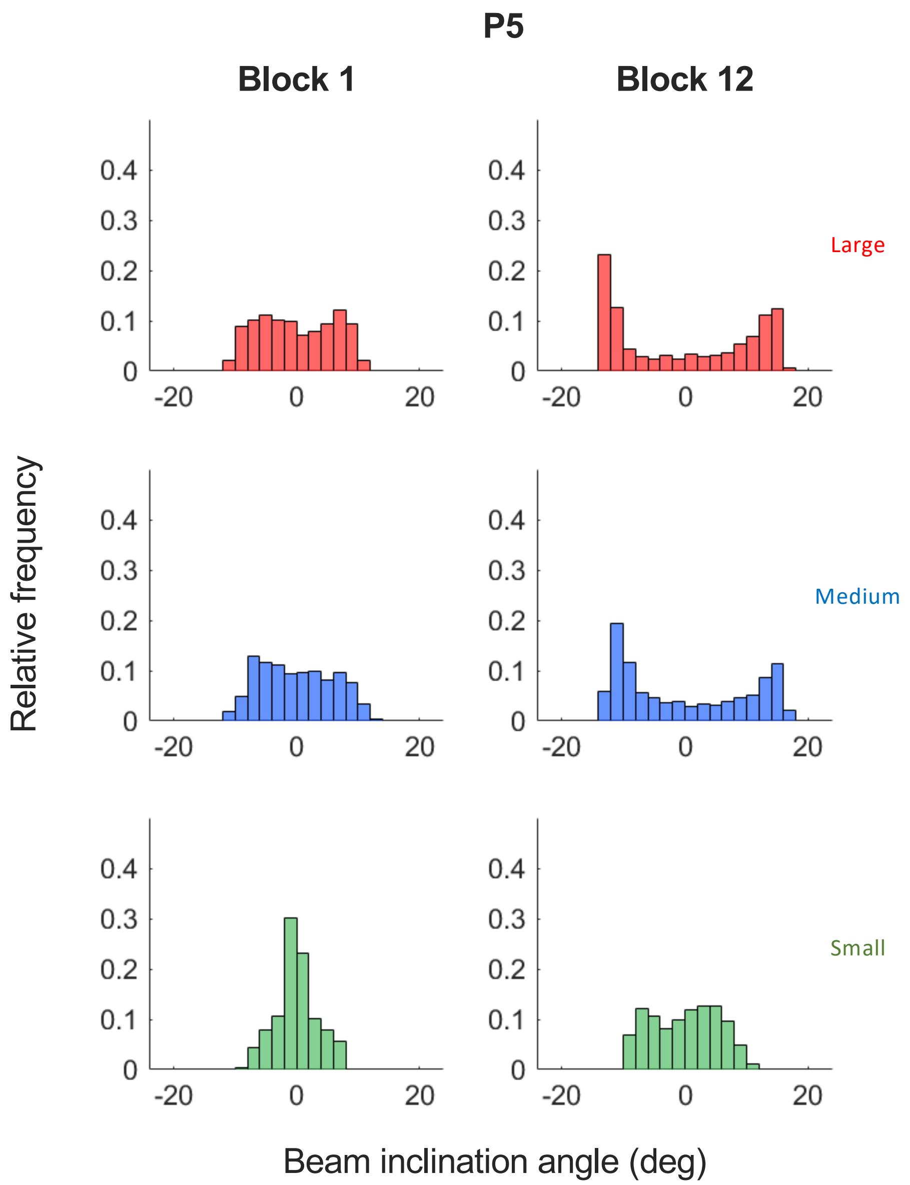
P5
Block 1
Block 12
Large
Medium
Small
Relative frequency
0.4
0.3
0.2
0.1
0
-20
0
20
Beam inclination angle (deg)

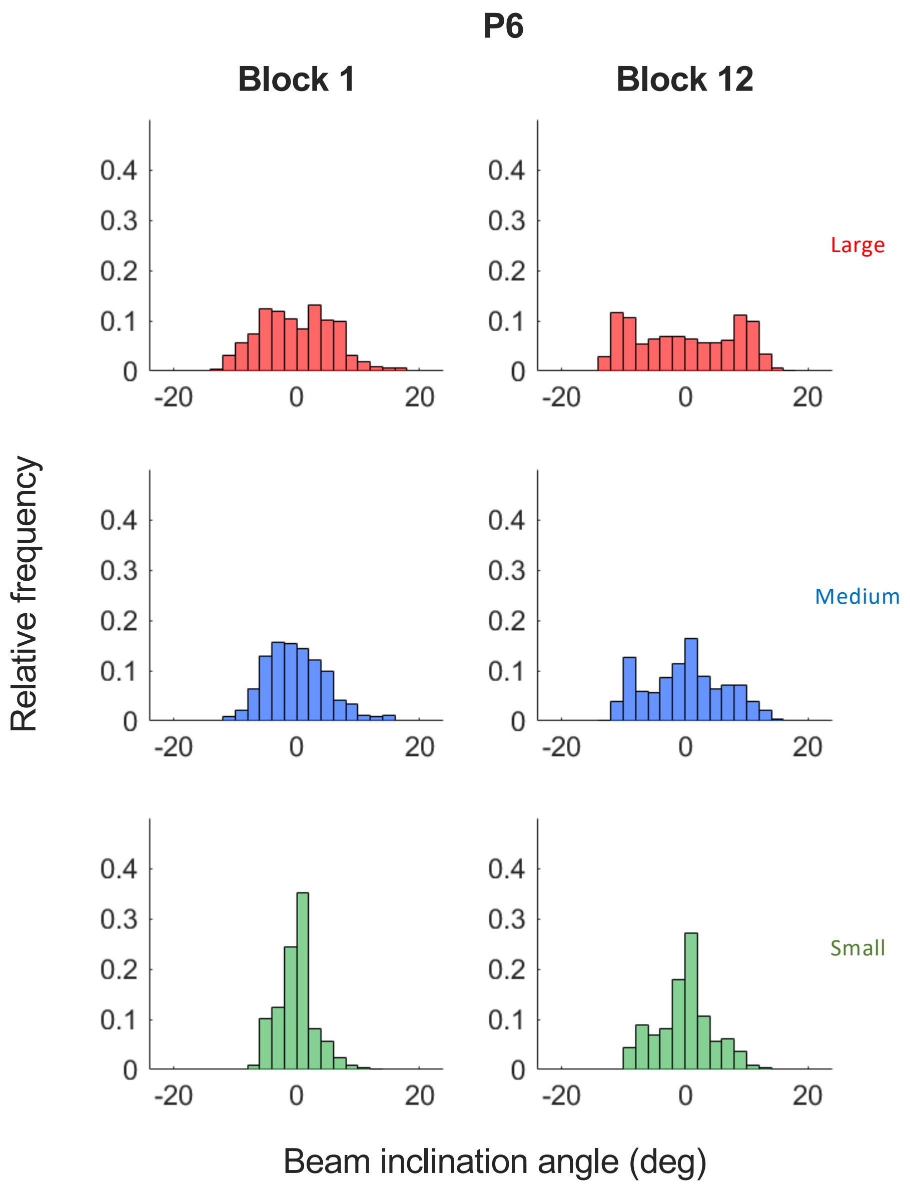
P6
Block 1
Block 12
Large
Medium
Small
Relative frequency
0.4
0.3
0.2
0.1
0
-20
0
20
Beam inclination angle (deg)

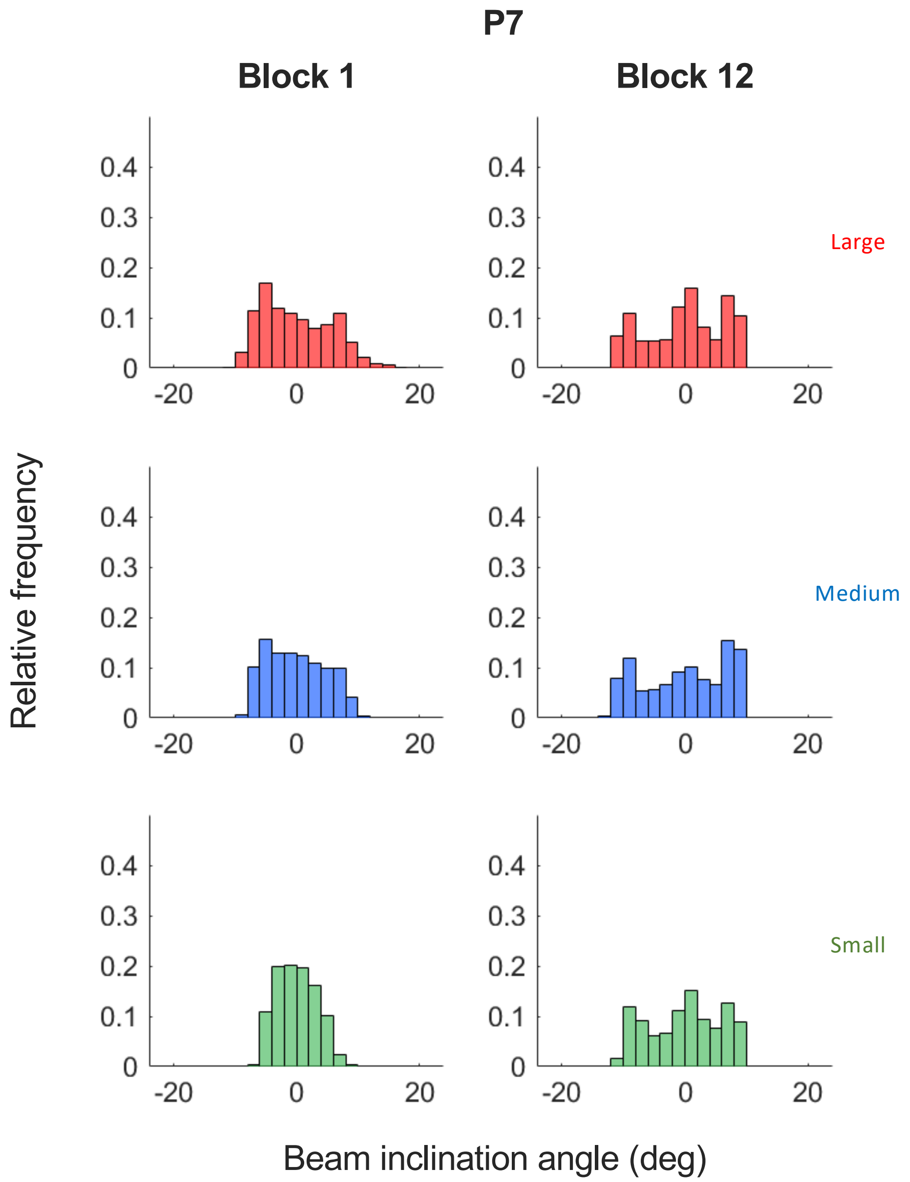
P7
Block 1
Block 12
Large
Medium
Small
Relative frequency
0.4
0.3
0.2
0.1
0
-20
0
20
Beam inclination angle (deg)

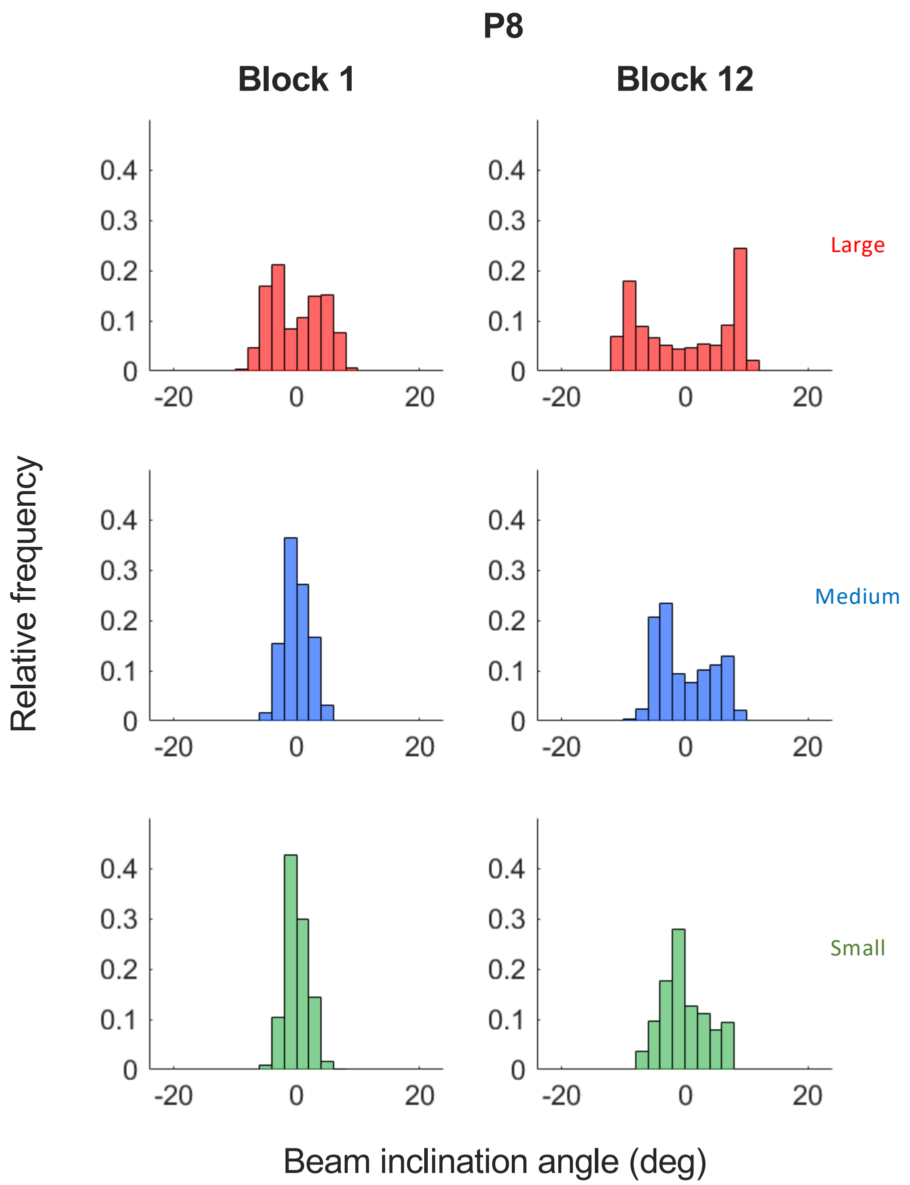
P8
Block 1
Block 12
Large
Medium
Small
Relative frequency
Beam inclination angle (deg)
0.4
0.3
0.2
0.1
0
-20
0
20

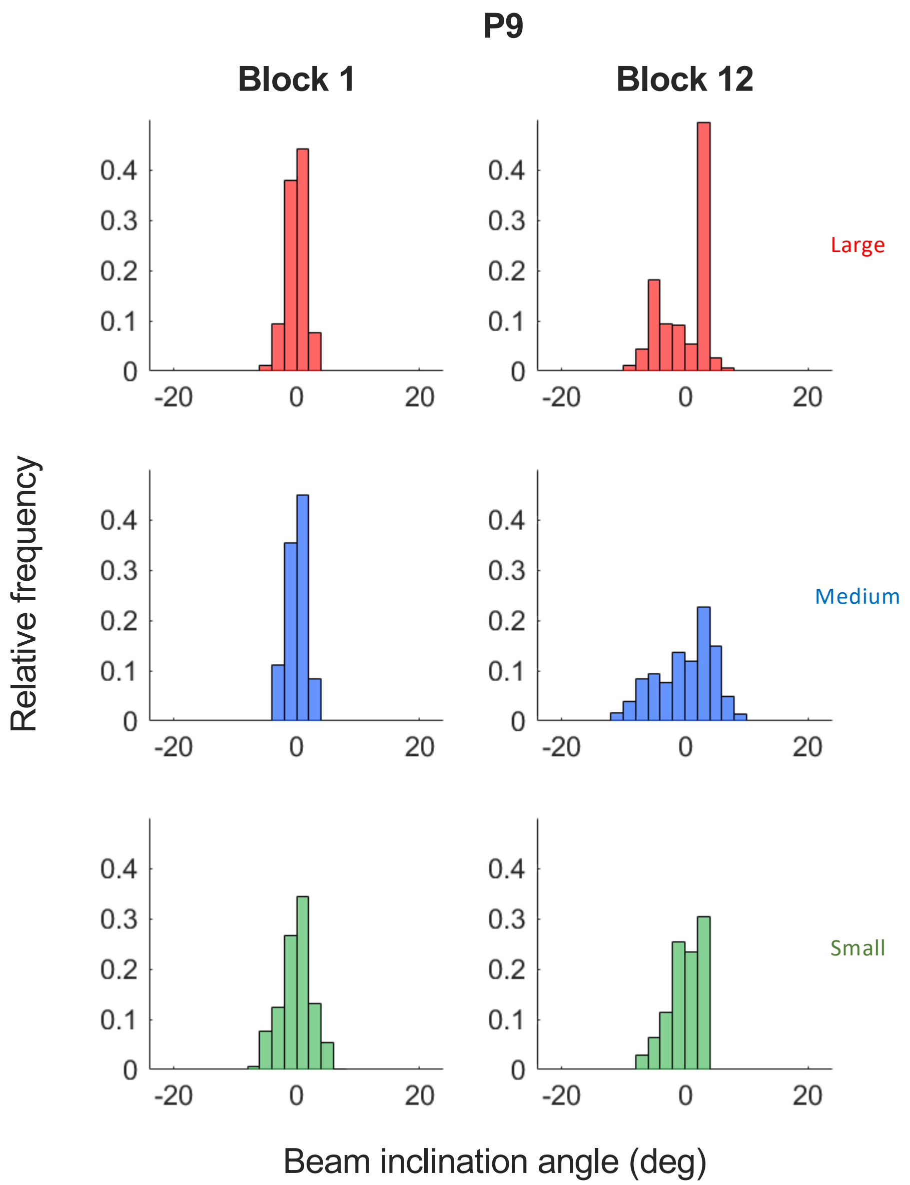
P9
Block 1
Block 12
Large
Medium
Small
Relative frequency
Beam inclination angle (deg)
0.4
0.3
0.2
0.1
0
-20
0
20

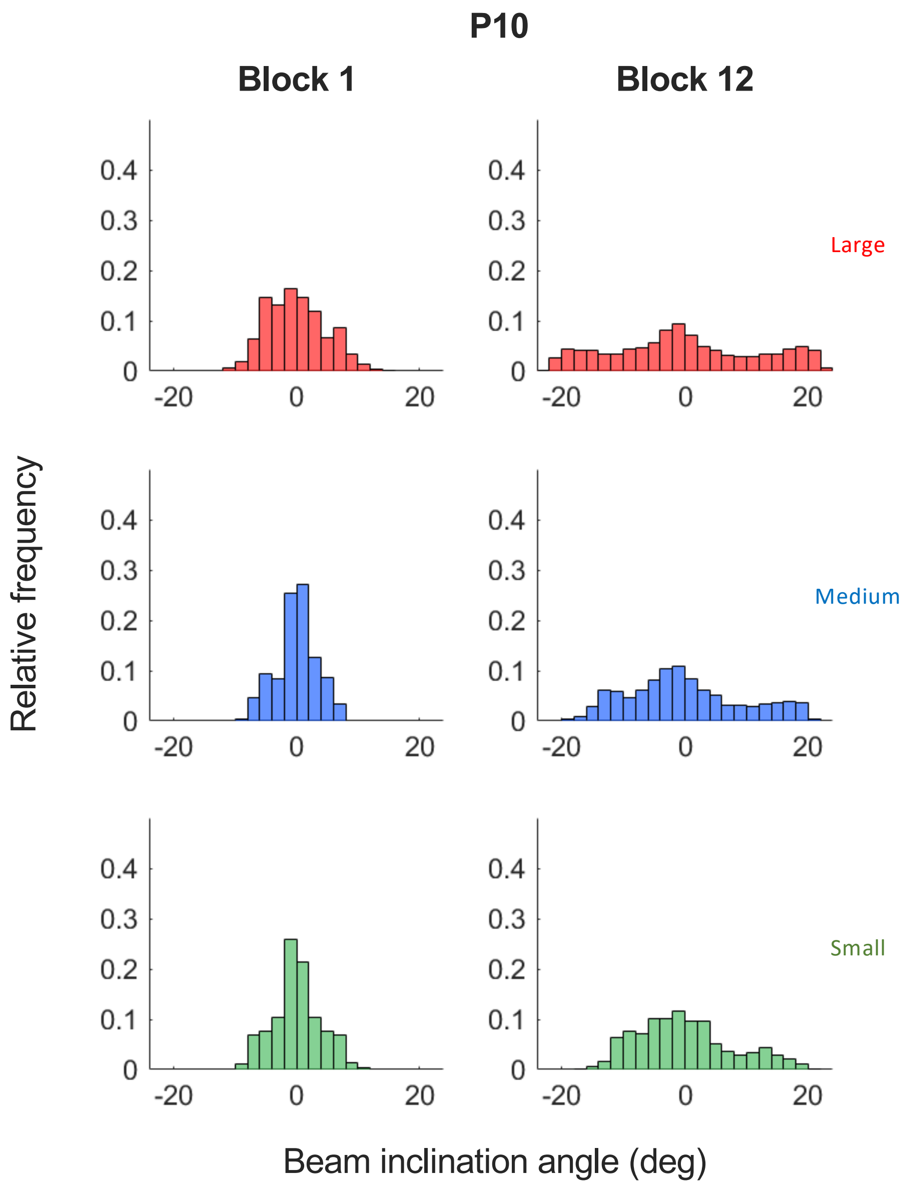
P10
Block 1
Block 12
Large
Medium
Small
Relative frequency
Beam inclination angle (deg)
0.4
0.3
0.2
0.1
0
-20
0
20

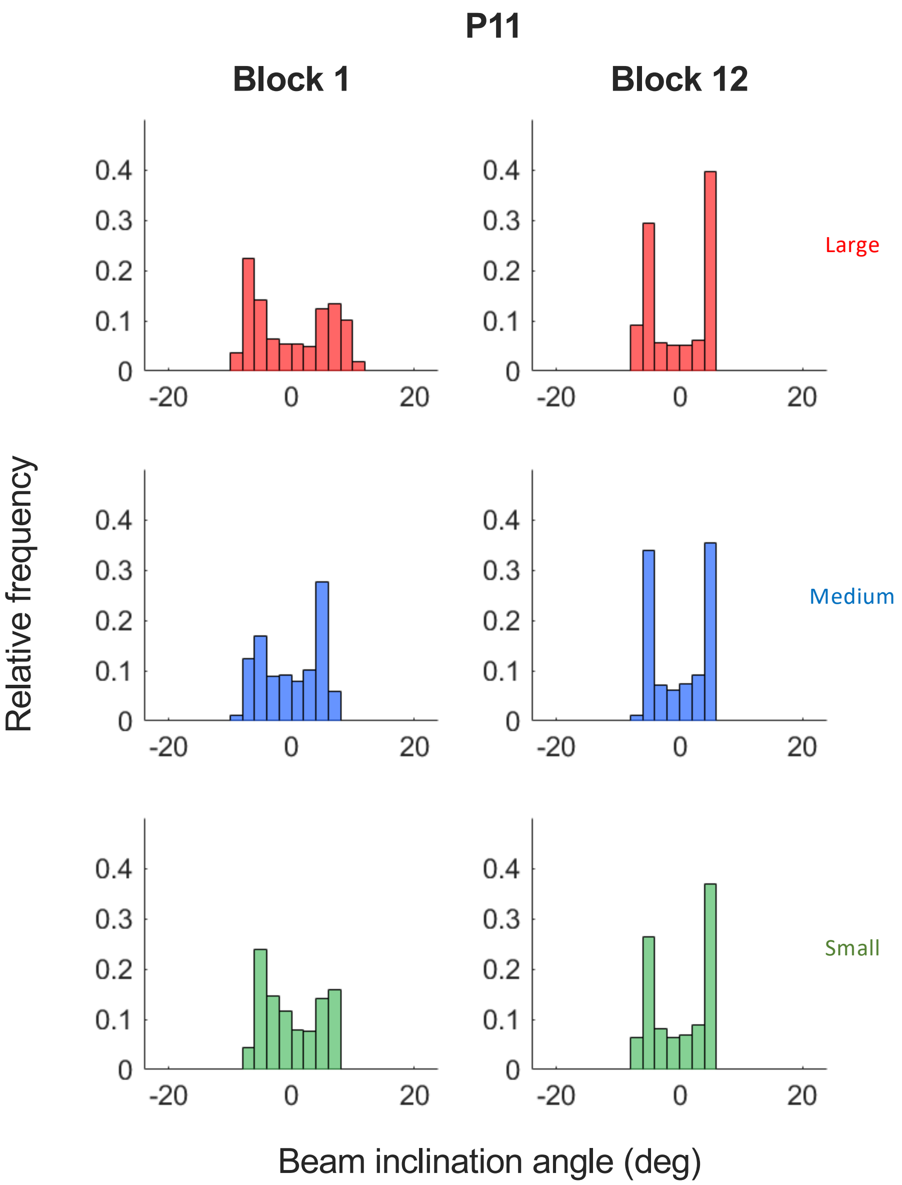
P11
Block 1
Block 12
Large
Medium
Small
Relative frequency
Beam inclination angle (deg)
0.4
0.3
0.2
0.1
0
-20
0
20

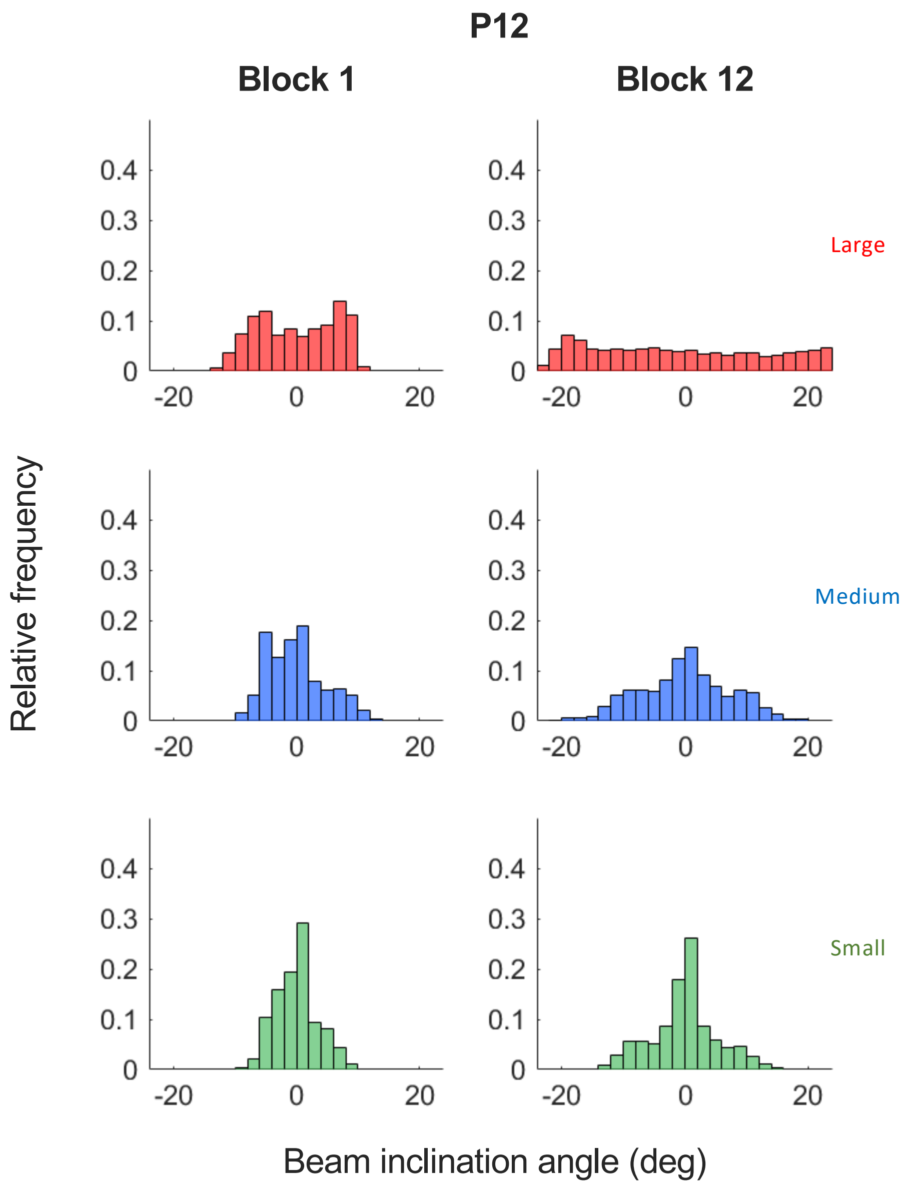

P12
Block 1
Block 12
Large
Medium
Small
Relative frequency
Beam inclination angle (deg)
0.4
0.3
0.2
0.1
0
-20
0
20

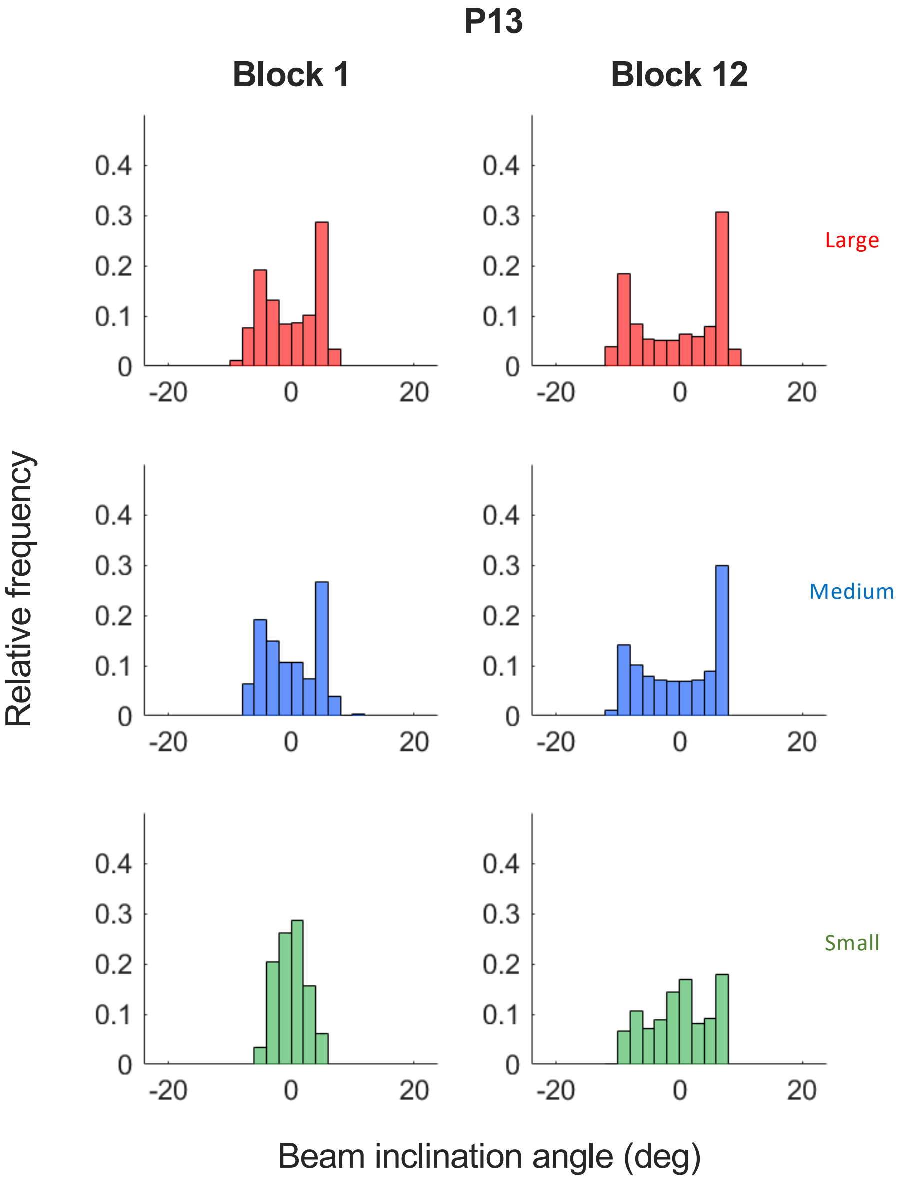

P13
Block 1
Block 12
Large
Medium
Small
Relative frequency
Beam inclination angle (deg)
0.4
0.3
0.2
0.1
0
-20
0
20

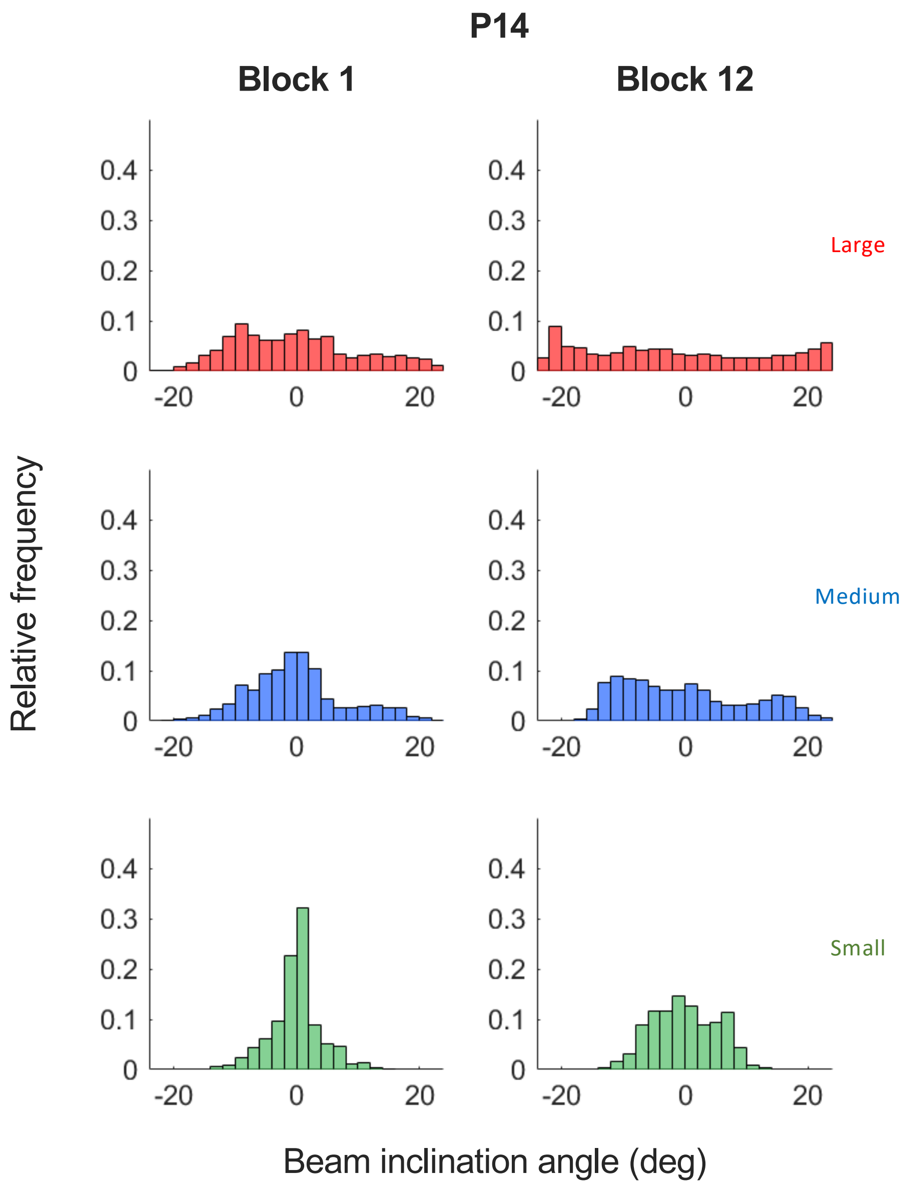
P14
Block 1
Block 12
Large
Medium
Small
Relative frequency
Beam inclination angle (deg)
0
0.1
0.2
0.3
0.4
-20
0
20

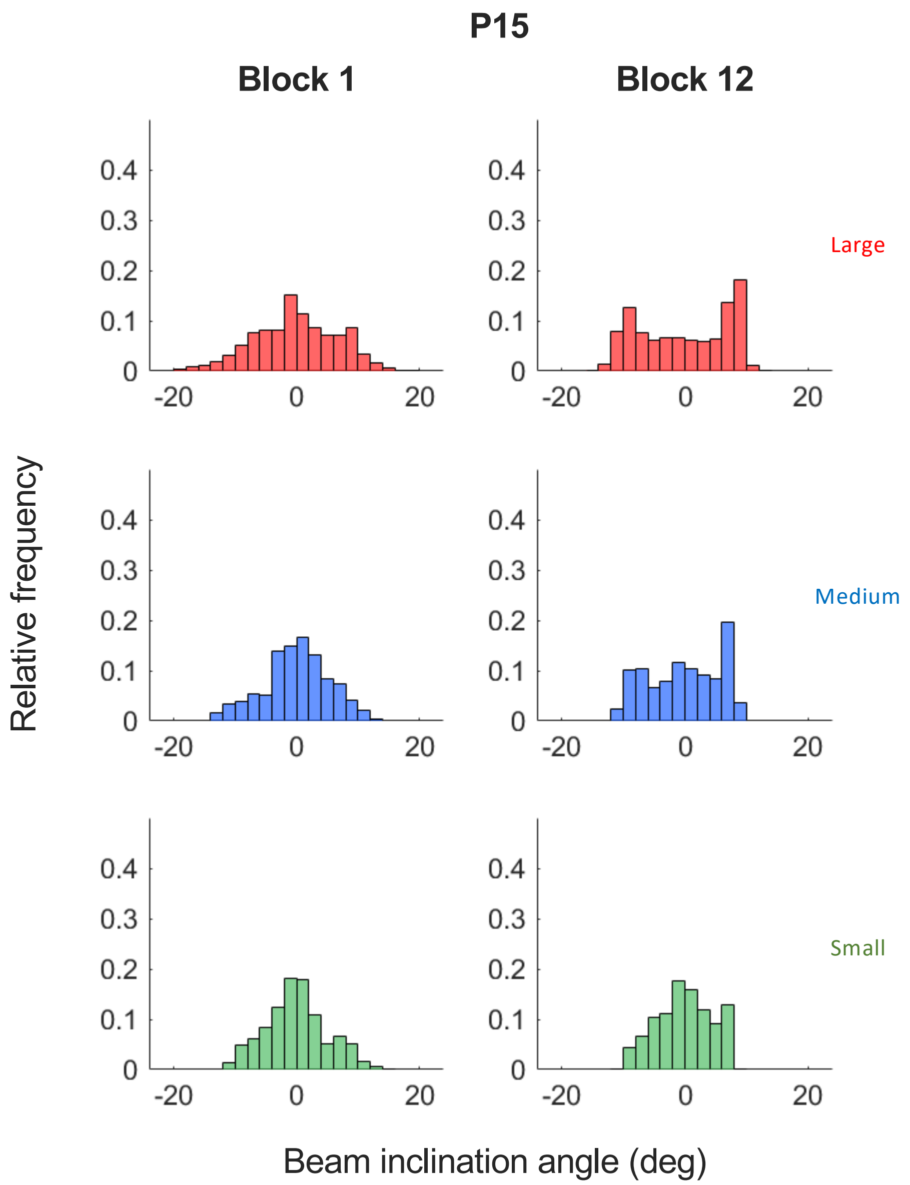
P15
Block 1
Block 12
Large
Medium
Small
Relative frequency
Beam inclination angle (deg)
0.4
0.3
0.2
0.1
0
-20
0
20

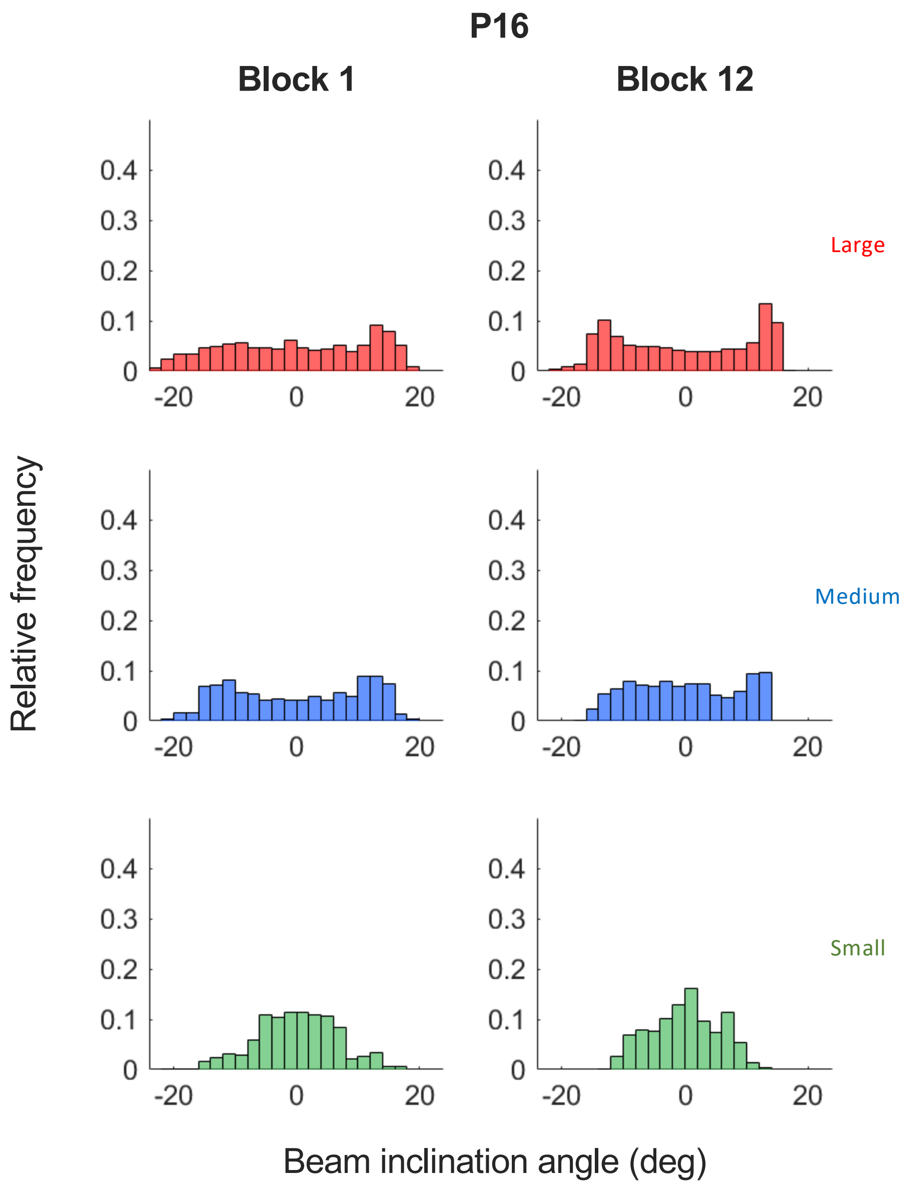
P16
Block 1
Block 12
Large
Medium
Small
Relative frequency
Beam inclination angle (deg)
0.4
0.3
0.2
0.1
0
-20
0
20
